# Contribution of schizophrenia polygenic burden to longitudinal phenotypic variance in 22q11.2 deletion syndrome

**DOI:** 10.1038/s41380-022-01674-9

**Published:** 2022-06-29

**Authors:** Maris Alver, Valentina Mancini, Kristi Läll, Maude Schneider, Luciana Romano, Estonian Biobank Research Team, Lili Milani, Mari Nelis, Reedik Mägi, Tõnu Esko, Andres Metspalu, Reedik Mägi, Emmanouil T. Dermitzakis, Stephan Eliez, Alexandre Reymond

**Affiliations:** 1 Department of Genetic Medicine and Development, University of Geneva School of Medicine, Geneva, Switzerland; 2 Estonian Genome Center, Institute of Genomics, University of Tartu, Tartu, Estonia; 3 Center for Integrative Genomics, University of Lausanne, Lausanne, Switzerland; 4 Developmental Imaging and Psychopathology Laboratory, University of Geneva School of Medicine, Geneva, Switzerland; 5 Clinical Psychology Unit for Intellectual and Developmental Disabilities, Faculty of Psychology and Educational Sciences, University of Geneva, Geneva, Switzerland; 6 Estonian Genome Center, Institute of Genomics, University of Tartu, Tartu, Estonia

**Keywords:** Genetics, Neuroscience, Schizophrenia, Predictive markers

## Abstract

While the recurrent 22q11.2 deletion is one of the strongest genetic risk factors for schizophrenia (SCZ), variability of its associated neuropsychiatric endophenotypes reflects its incomplete penetrance for psychosis development. To assess whether this phenotypic variability is linked to common variants associated with SCZ, we studied the association between SCZ polygenic risk score (PRS) and longitudinally acquired phenotypic information of the Swiss 22q11.2DS cohort (*n* = 97, 50% females, mean age 17.7 yr, mean visit interval 3.8 yr). The SCZ PRS with the best predictive performance was ascertained in the Estonian Biobank (*n* = 201,146) with LDpred. The infinitesimal SCZ PRS model showed the strongest capacity in discriminating SCZ cases from controls with one SD difference in SCZ PRS corresponding to an odds ratio (OR) of 1.73 (95% CI 1.57–1.90, *P* = 1.47 × 10^−29^). In 22q11.2 patients, random-effects ordinal regression modelling using longitudinal data showed SCZ PRS to have the strongest effect on social anhedonia (OR = 2.09, *P* = 0.0002), and occupational functioning (OR = 1.82, *P* = 0.0003) within the negative symptoms course, and dysphoric mood (OR = 2.00, *P* = 0.002) and stress intolerance (OR = 1.76, *P* = 0.0002) within the general symptoms course. Genetic liability for SCZ was additionally associated with full scale cognitive decline (β = –0.25, *P* = 0.02) and with longitudinal volumetric reduction of the right and left hippocampi (β = –0.28, *P* = 0.005; β = –0.23, *P* = 0.02, respectively). Our results indicate that the polygenic contribution to SCZ acts upon the threshold-lowering first hit (i.e., the deletion). It modifies the endophenotypes of 22q11.2DS and augments the derailment of developmental trajectories of negative and general symptoms, cognition, and hippocampal volume.

## Introduction

Genomic studies have established the importance of copy number variants (CNVs) in rare disease aetiology, and particularly as causal factors for neurodevelopmental disorders [1]. Despite conferring substantial risk for severe outcomes, CNVs often exhibit incomplete penetrance and wide variability in clinical manifestations [2–5], suggesting complex mechanisms for disease liability.

One of the most common genomic rearrangements is a recurrent hemizygous deletion on chromosome 22q11.2. In 90% of the cases, the deletion occurs *de novo* via non-allelic homologous recombination of low copy repeats [6]. In addition to being associated with characteristic physical features, cognitive deficits, heart problems, and neuropsychiatric symptoms [6–9], it is one of the most common aetiology for schizophrenia (SCZ) with a penetrance of 25–40% [10, 11]. Such incomplete penetrance could be associated with environmental and/or genetic factors as proposed for neurodevelopmental manifestations linked to 16p11.2 rearrangements [12]. Multiple lines of evidence support this “multiple hit” model, whereby secondary hits (i.e., modifying genetic factors) in addition to the threshold-lowering first hit (i.e., 22q11.2 deletion) modulates the clinical outcomes [13, 14]. While in rare cases the potential modifier effects of the 22q11.2 deletion syndrome (22q11.2DS) were attributed to variable deletion size [15], hemizygosity of single nucleotide variants (SNVs) on the intact allele [16, 17], and additional rare CNVs [18], an increasing burden of evidence suggests that common allelic variation of SNVs pertaining to SCZ biology could explain variability in neuropsychiatric symptoms of 22q11.2DS [16, 19], specifically psychosis development and cognitive decline [20, 21].

Trait-associated SNV effects pooled into a single number, namely a polygenic risk score (PRS) could capture a meaningful proportion of phenotypic variance (e.g., 7.7% for SCZ phenotype [22]) and thus facilitates the estimation of genetic liability for a trait of interest as well as for endophenotypes and biologically overlapping outcomes. SCZ PRS has been associated with prodromal motor deficits [23], cognitive ability [24, 25], and disorganized symptoms in the general population [25], negative symptoms and anxiety in adolescence [26], and greater illness severity and worse cognition within a psychosis cohort [27]. SCZ PRS was also linked with decreased total brain volume and cortical thickness [28, 29], reduced neurite density index, especially in the thalamus, basal ganglia, and hippocampus [30], thinner frontotemporal cortices and a smaller hippocampal subfield volume [31], as well as with impaired mnemonic hippocampal activity [32]. These results collectively substantiate the polygenic and complex nature of SCZ as well as lay the premise for investigating the contribution of its genetic load to phenotypic variability in 22q11.2DS.

Building upon previous findings [20, 21], we assessed the contribution of the SCZ polygenic burden on clinical symptoms associated with psychosis risk, cognitive ability, and brain imaging variables among longitudinally followed 22q11.2DS patients [33], a unique source for investigating phenotypic and molecular abnormalities specific to this disorder through time (Supplementary Fig. 1).

## Methods

### Overview of the 22q11.2DS cohort

#### Participants

Recruitment of participants within the Swiss 22q11.2DS longitudinal cohort [33] began in 2001 through word of mouth, community announcements and advertisements aimed at parents’ associations. The presence and extent of a 22q11.2 microdeletion was confirmed in all patients using quantitative fluorescent polymerase chain reaction. At first visit and during follow-up visits, individuals with 22q11DS underwent magnetic resonance imaging (MRI) acquisition and a broad set of clinical and cognitive tests (Table 1). Five individuals carried a smaller 1.5 Mb (LCR22A to LCR22B) deletion, confirming that the recurrent deletion of 3 Mb (LCR22A to LCR22D) is the predominant one among 22q11.2DS patients [14, 34–36]. As the key 22q11.2DS phenotype has been shown to result largely from the diminished LCR22A to LCR22B deletion gene dosage [37, 38], we included these five individuals in downstream analyses, but accounted for them (see below in Association testing section in Methods). Written informed consent was obtained from participants and/or their parents. The study was approved by the cantonal ethics committee and conducted according to the Declaration of Helsinki.

**Table 1:** Characteristics of the Swiss 22q11.2DS longitudinal cohort.

Characteristic	Count
N of subjects (females)	97 (49)
Age range	6–44
Mean age (SD)	17.67 (6.3)
Mean age at first visit (SD)	15.0 (6.66)
Mean interval between visits (SD)	3.80 (1.07)
N of subject with SIPS, MRI, IQ data	84
N of subject with only MRI and IQ data	9
N of subject with only SIPS data	4

*SD* standard deviation, *SIPS* Structured Interview for Psychosis-Risk Syndromes, *MRI* magnetic resonance imaging, *IQ* intelligence quotient score.

#### Psychiatric assessment

The presence of attenuated psychotic symptoms was evaluated at each visit by an expert psychiatrist using the Structured Interview for Psychosis-risk Syndromes (SIPS) [39], which is a well-validated diagnostic tool for assessing psychotic symptoms for 22q11.2DS patients as shown in previous studies [40, 41]. Item scores in each domain of SIPS (positive, negative, disorganized, and general symptoms) on a 7-point scale ranging from zero (absent) to maximum six (extreme/severe) were used for downstream analyses. A psychosis positive variable was derived in case one or more of the items in the positive symptoms category had a score ≥3. Together with time and frequency criteria, this intensity threshold has been proven to be the most sensitive at detecting prodromal risk syndromes [42].

#### Intellectual functioning

At each visit, all participants underwent the administration of the Wechsler Adult Intelligence Scale (WAIS-III and WAIS-IV) [43] or the Wechsler Intelligence Scale for Children (WISC-III and WISC-IV) [44] to evaluate general intelligence and reasoning abilities over time. For the purposes of this study, we analysed full-scale intelligence quotient (FSIQ), and the subscales of verbal IQ (VIQ) and performance IQ (PIQ). While different versions of the test (version III or IV) were used between participants over the years to fit the longitudinal design as described in previous studies [45, 46], the same version was kept for each participant across visits. The type of test was used as a covariate in analyses with IQ measurements.

#### MRI acquisition

Due to the wide timespan of the study, MRI scans were acquired with three different scanners: 1.5 T Philips Intera scanner was used for the first 20 scans, 3 T Siemens Trio for the subsequent 94 scans, and 3 T Siemens Prisma for the remaining 93 scans. T1-weighted images were acquired at the Center for Biomedical Imaging in Geneva with a three-dimensional volumetric pulse. 1.5 T scanner parameters were TR = 35 ms, TE = 6 ms, flip angle = 45°, NEX = 1, matrix size = 256 × 192, field of view = 24 cm^2^, slice thickness = 1.5 mm, and 124 slices. The parameters for both 3 T scanners were TR = 2500 ms, TE = 3 ms, flip angle = 8°, acquisition matrix = 256 × 256, field of view = 23.5 cm, slice thickness = 3.2 mm, and 192 slices. To minimize any potential confounder effects, the scanner model was used as a covariate in statistical analyses using neuroimaging measurements.

#### T1-weighted images analysis

T1-weighted images underwent fully automated image processing with FreeSurfer v6.0, comprising skull stripping, intensity normalization, reconstruction of the internal and external cortical surface and parcellation of subcortical brain regions [47]. Cortical thickness was computed as the shortest distance between the white and the pial cortical surfaces [48, 49] and surface area was measured at the grey/white matter boundary. Average measures of cortical thickness and surface area were extracted from 68 regions based on the Desikan parcellation [50]. An automated segmentation technique published with FreeSurfer v6.0 [51] was employed to obtain the volume of the whole hippocampus and seven relevant subfields, including CA1, CA2/3, CA4, GC-DG, ML, tail, and subiculum. All the obtained images were visually inspected and excluded from downstream analysis if the quality of the segmentation was sub-optimal as explained in detail in Mancini et al. [52].

#### Genotyping

One hundred and twenty-two individuals whose DNA samples were available within the Swiss 22q11.2DS cohort, were subjected for whole-genome genotyping with the Illumina Global Diversity Array v1. Quality control was carried out with PLINK v2.0 [53] (webpage: https://www.cog-genomics.org/plink/2.0/) using the following criteria: (i) exclusion of individuals with genotype call rate <95%; (ii) exclusion of single nucleotide variants (SNVs) with call rate <95%, Hardy-Weinberg equation (HWE) < 1e-4, minor allele frequency (MAF) < 0.01, and with A/T or G/C alleles to avoid strand issues; (iii) removal of outliers who deviated ± 3 standard deviations from the samples’ heterozygosity rate mean, and (iv) verification that the data did not contain closely related individuals (PI_HAT > 0.2) and that phenotype and genotype sex matched. Of first-degree relatives, one member of each related pair was excluded, preferentially retaining samples that had more complete phenotype data. Deletion carrier status was confirmed with bcftools cnv calling plugin (https://samtools.github.io/bcftools/howtos/cnv-calling.html) [54]. The 1000 Genome Project data [55] was used as reference to exclude samples that showed differential ancestral background than European based on principal component analysis (PCA) (Supplementary Fig. 3). Genetic principal components were calculated with QTLtools pca mode using variant sites separated by 5000 base pairs [56] (webpage: https://qtltools.github.io/qtltools/). Haplotype Reference Consortium reference panel [57] (webpage: http://www.haplotype-reference-consortium.org/) was used for array imputation with the following parameters: build hg19, reference panel apps@hrc-r1.1, population European, phasing eagle. After imputation, SNVs with low imputation quality score R2 < 0.3, HWE *p* < 1e-6 and MAF < 0.05 were filtered out. The final quality controlled SNV set contained 6,462,855 biallelic SNVs for 103 individuals. Six individuals were further excluded as no phenotype data was available either due to their young age for completing SIPS or due to sub-optimal MRI data, thus reducing the sample set to 97 patients.

### Derivation of the polygenic risk score for schizophrenia (SCZ PRS)

For constructing and identifying the SCZ PRS with the best predictive performance, we used the summary statistics from the SCZ genome-wide association analysis (GWAS) wave3 by the Psychiatric Genomics Consortium conducted primarily on samples of European ancestry [22], phenotype and genotype data collected within the Estonian Biobank (EstBB) [58] and the LDpred algorithm [59].

EstBB is a population-based biobank in Northern Europe, comprising 201,146 individuals aged ≥18 years. All biobank participants have signed a broad informed consent form, which allows continuous updating of epidemiologic data through periodical linking to national electronic repositories (hospital databases, national registries), and recontacting of participants. Medical history and health status are recorded according to the International Classification of Diseases, Tenth Revision (ICD-10 codes) [58]. EstBB participants have been genotyped using Illumina Global Screening Arrays with quality control conducted according to best practices (exclusion of individuals with call rate <95%, mismatch of genotype and phenotype sex, exclusion of SNVs with call rate <95%, HWE *p* < 1e-4, MAF < 1%). Pre-phasing was carried out with Eagle v2.3 [60] and imputation with Beagle v5 (28Sep18.79)8 [61] using the population specific imputation reference panel built from 2297 whole genome sequencing samples [62].

Genome-wide SCZ PRSs were constructed with LDpred, a Bayesian approach that applies a continuous shrinkage model to modify effect sizes based on the strength of each variant’s association in the GWAS and the underlying linkage disequilibrium (LD) structure [59]. We started with 7,585,078 SNVs for which the summary statistics level data from the SCZ GWAS wave3 was available [22] (https://www.med.unc.edu/pgc/download-results). The EstBB SNV content was (i) filtered for the quality controlled SNV content captured in the Swiss 22q11.2DS genotype data to account for the uniform set of SNVs in both datasets (resulted in 5,459,498 SNVs), (ii) filtered for the quality controlled SNV content (MAF > 0.01 and imputation quality score >0.8) in EstBB data (resulted in 5,235,126 SNVs), and (iii) clumped for maximum LD between SNV to reduce multicollinearity dimensions (r2 = 0.99; resulted in 2,473,370 SNVs). Ten different SCZ PRSs were derived by varying the fraction of causal SNVs (infinitesimal, *p* ≤ 1, *p* ≤ 0.3, *p* ≤ 0.1, *p* ≤ 0.03, *p* ≤ 0.01, *p* ≤ 0.003, *p* ≤ 0.001, *p* ≤ 0.0003, and *p* ≤ 0.0001) and using the EstBB LD reference panel to account for LD between SNVs.

For testing and validating the SCZ PRSs in EstBB, we excluded EstBB participants whose data was included in the SCZ GWAS wave3, one member per related individual pairs (PI_HAT > 0.2) and individuals with non-European ancestry in reference to 1000 Genome Project samples [55]. SCZ cases were defined using two sub-group criteria based on ICD-10 codes in electronic health records: (i) relaxed “Schizophrenia Spectrum Disorder” diagnosis (ICD-10 F2* “Schizophrenia, schizotypal, delusional, and other non-mood psychotic disorders” category; resulted in 1,356 SCZ cases), and (ii) strictly “Schizophrenia” diagnosis (ICD-10 code F20.* “Schizophrenia” category; resulted in 572 SCZ cases). Based on the consultation with practising Estonian psychiatrists to define the definition of SCZ diagnosis using ICD-10 codes reported in the national healthcare system, we opted for testing the SCZ PRSs using two SCZ definition groups to account for the following factors: (1) loss of power due to volunteer-based recruitment resulting in low number of strictly SCZ cases (i.e., considering ICD-10 F20.*), (2) possible increase in noise when relaxing SCZ diagnosis criteria (i.e., considering ICD-10 F2*). We considered SCZ cases with at least one report of an ICD-10 code for Schizophrenia Spectrum Disorder/Schizophrenia given by a psychiatrist or a neurologist and excluded individuals carrying SCZ diagnosis as a comorbid condition only or diagnosed by a non-specialist. EstBB participants without ICD-10 F2* were considered as controls (*n* = 108,201). Individuals with mania (ICD-10 F30.* “Manic episodes” category) and bipolar disorder (F31.* “Bipolar disorder” category) were excluded from all sets given the considerable genetic overlap between these psychiatric disorders and SCZ [63] (further information in the Supplementary Note).

Next, two-thirds of the EstBB cohort were allocated into a testing set (71,412 controls; 894 SCZ cases with F2* diagnosis, and 377 SCZ cases with F20* diagnosis) and one-third into a validation set (36,789 controls; 462 SCZ cases with F2* diagnosis and 195 SCZ cases with F20* diagnosis) for identifying and validating the best performing PRS, respectively (overview of the characteristics of the testing and validation sets are outlined in Supplementary Table 1). All ten SCZ PRSs retrieved with the LDpred method were computed for all individuals with STEROID v0.1.1 (https://genomics.ut.ee/en/tools) by multiplying the genotype dosage of each risk allele for each SNV by its respective weight and then summing across all SNVs into a score. For determining the best predicting PRS, we considered ten standardized SCZ PRSs separately and used a logistic model with diagnosis status (SCZ case or control) as a dependent variable and sex, age, and five genotype PCs as covariates. The model with the highest odds ratio was selected for replication in the validation set. The score with the best discriminative capacity in the validation set was additionally assessed based on maximal area under the receiver-operator curve (AUC) for considered logistic regression models using R/*pROC* package [64] and using R/*survival* package [65] (latter was used to account for age effect using left truncation and right censoring). Individual level data analysis was carried out under ethical approval 1.112/624 from the Estonian Committee on Bioethics and Human Research (Estonian Ministry of Social Affairs) and data release N05 from EstBB.

### Association testing in the 22q11.2DS cohort

The SNVs and their adjusted weights of the best performing SCZ PRS (i.e., infinitesimal SCZ PRS model) were used for calculating the SCZ PRS for 22q11.2DS patients with STEROID v0.1.1 (https://genomics.ut.ee/en/tools) and standardized such that it followed a normal distribution with mean 0 and standard deviation 1 (Supplementary Fig. 6a).

To test for an association between 19 SIPS variables (ordered factor dependent variables) and SCZ PRS, we used ordinal logistic regression implemented in R/*mass* package (polr function) [66] for cross-sectional analysis and random-effects ordinal regression implemented in R/*ordinal* package (clmm function) [67] for longitudinal analysis. In the latter approach we considered each participant having SIPS variable data captured at multiple timepoints as random effects. Age, sex and first three genotype PCs were accounted for as covariates in cross-sectional analysis, while age^2^ was added in longitudinal analysis. SIPS data acquired at the timepoint in which the age was the closest to the median age of the 22q11.2DS cohort (median 16.43, mean 17.30, SD 4.91) were considered in cross-sectional analysis. Violation of proportional odds assumption was tested with Brant test that allows to assess whether the observed deviations from ordinal logistic regression model are larger than what could be attributed to chance alone using R/*brant* package [68]. The probabilities for each model with SIPS variables are given in Supplementary Table 2. No evidence for violating the proportional odds assumption was found (*p* > 0.05). To correct for multiple testing, false discovery rate (FDR) and Bonferroni correction were applied for cross-sectional and longitudinal analysis, respectively, accounting for 19 tests (R*/qvalue* package [69]). Given the small sample size, we additionally applied bootstrapping for each longitudinally tested model and carried out 1000 runs using sampling with replacement. Next, we considered the mean of p-values across bootstrapping runs for each item and determined model ranking based on the proportion (%) how many times the model was deemed significant at nominal p-value of <0.05 across 1000 bootstrapping runs. Items that surpassed Bonferroni correction and that were supported by bootstrapping were deemed as significant. SIPS variables were available for 88 individuals from 213 timepoints. To test whether SCZ PRS was correlated with positive or negative symptoms at different ages, we divided the cohort into two subsets using 18 years as the cut-off and carried out association testing cross-sectionally and longitudinally. A “positive symptoms” variable and a “negative symptoms” variable were derived by pooling values across respective category items. In cross-sectional analysis, the mean age of the younger sub-group (<18 years, *n* = 54) was 14.51 with median 14.67 and SD 2.16; and the mean age of the older sub-group (≥18 years, *n* = 34) was 21.72 with median 20.39 and SD 4.81. In longitudinal analysis, data of 76 individuals from 111 timepoints and 49 individuals from 102 timepoints were available for the younger and for the older sub-group, respectively.

Linear regression was used to test for an association between SCZ PRS and IQ and MRI variables cross-sectionally using data from the timepoint in which the age was the closest to the median age of the cohort (median 16.43, mean 17.30, SD 4.91). SCZ PRS was regressed on age, IQ test type/MRI scanner, and first three genotype PCs. Next, we used longitudinal data for associating cognitive function and brain imaging variables captured at multiple timepoints with SCZ PRS. To this end, we used linear mixed modelling implemented in R/*lme4* package (lmer function) [70] to account for within-subject correlations by including a random intercept for each subject and considered age, age^2^, IQ test type/MRI scanner, and first three genotype PCs as covariates. For cognition, we first tested full scale IQ independently and then conducted a sub-analysis by considering verbal IQ and performance IQ measurements. For hippocampus, we carried out a secondary, region of interest analysis and considered fourteen volumetric hippocampal subfield variables. FDR correction [69] was applied for multiple testing. IQ measurements and brain imaging variables were available for 93 individuals from 212 timepoints, and 93 individuals from 207 timepoints, respectively, and were standardized such that these followed normal distribution with mean 0 and standard deviation 1.

To account for the five individuals with smaller 1.5 Mb deletion, we conducted a sensitivity analysis for all neuropsychiatric phenotypes considering 3 Mb deletion carriers only. While the test statistics show attenuation due to reduced power, these followed the same trend as in the main analyses (Supplementary Table 6).

Statistical analyses were conducted with R software version 3.6.2 [71].

## Results

### Swiss 22q11.2DS longitudinal cohort

Ninety-seven genotyped individuals (49 females) aged from 6 to 44 years (mean = 17.67, SD = 6.30) with a molecularly confirmed diagnosis of 22q11.2DS were included in the present study. Each participant was phenotypically assessed at an average of 2.2 timepoints (range = 1–5). Mean age at first visit was 15 years (SD = 6.66) and mean time interval between visits was 3.8 years (SD = 1.07; Table 1, Supplementary Fig. 2).

### Identification of the best performing polygenic risk score for schizophrenia

**Fig. 1:**
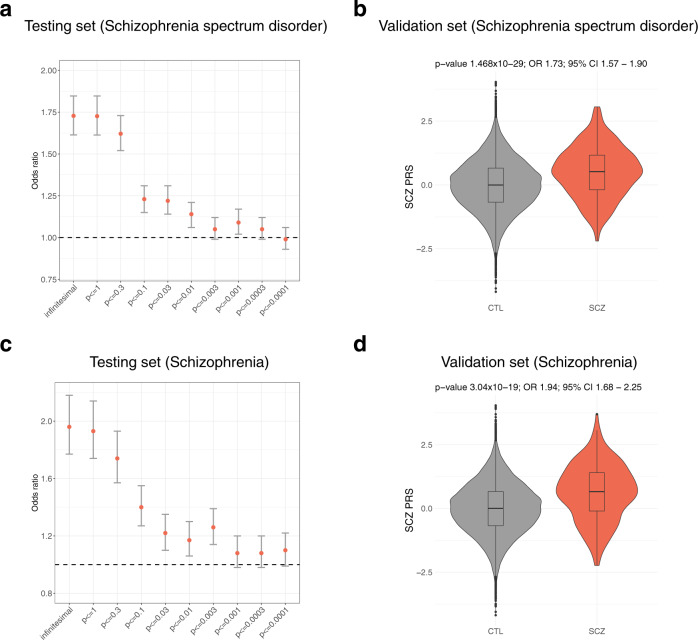
Predictive ability of SCZ PRS in EstBB. Odds ratios and 95% confidence intervals for ten SCZ PRSs in the testing set (**a**, **c**) and boxplots of the best performing SCZ PRS (infinitesimal model) in SCZ cases and controls (CTL) in the validation set (**b**, **d**). Schizophrenia Spectrum Disorder diagnosis and strictly Schizophrenia diagnosis were used for determining SCZ cases in the upper (**a**, **b**) and lower panels (**c**, **d**), respectively.

Using LDpred, we constructed ten candidate SCZ PRSs using summary statistics from the SCZ GWAS wave3 [22] and tested and validated their predictive performance in EstBB comprising 201,146 individuals of European ancestry [58]. Using a testing set of 462 Schizophrenia Spectrum Disorder cases and 71,412 controls (Supplementary Table 1), we showed that the infinitesimal model, i.e., all genetic variants deemed causal for SCZ, showed the strongest effect in discriminating SCZ cases from control subjects (Fig. 1a, b; Supplementary Fig. 4a). One SD difference in SCZ PRS corresponded to an odds ratio (OR) of 1.73 (95% confidence interval (CI) 1.57–1.90, *P* = 1.47 × 10^−29^). These results were in concordance with estimates when considering a lower number of SCZ cases determined with stricter SCZ diagnostic criteria (Fig. 1c, d; Supplementary Fig. 4b; Supplementary Table 1). The prediction accuracy for the infinitesimal model was additionally assessed using maximal area under the receiver-operator curve (AUC). For the model containing covariates only (sex, age, five population structure PCs), the AUC was 0.653. Adding SCZ PRS to the model increased the AUC to 0.703, resulting in a 5% increase (Supplementary Fig. 5). As age was the main predictor, we additionally determined the discrimination capacity of SCZ PRS between SCZ cases and controls at the same age. Harrell’s C statistic of the model with age as timescale and without SCZ PRS in the model was 0.58 (95% CI 0.51–0.64) and 0.68 (95% CI 0.54–0.81) when considering Schizophrenia Spectrum Disorder and strictly Schizophrenia cases, respectively, and with SCZ PRS in the model increased to 0.64 (95% CI 0.58–0.70) and to 0.77 (95% CI 0.68–0.85) using the respective SCZ diagnostic criteria groups. These results agree with prior findings underscoring high polygenicity for SCZ [22, 72] as well as with AUC estimates determined for SCZ PRS [73, 74]. The SNVs and their adjusted weights of the infinitesimal SCZ PRS model were used for calculating SCZ PRS for 22q11.2DS patients. No discordance in the distributions of SCZ PRS values between EstBB and Swiss 22q11.2DS samples was identified in agreement with previous data [21] (Supplementary Fig. 6b).

### Polygenic burden for schizophrenia and phenotypic variance of 22q11.2DS

**Fig. 2:**
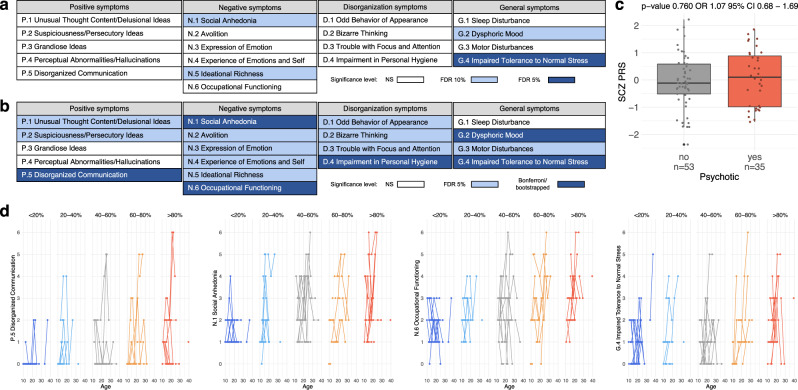
SCZ PRS association with SIPS variables. Overview of (**a**) cross-sectional and (**b**) longitudinal analyses results for SCZ PRS and SIPS variables with colour darkness indicating association strength after multiple correction, NS—not significant (white); FDR 5% (light blue) and Bonferroni/bootstrapped—associations that surpassed Bonferroni correction and were supported by bootstrapping (dark blue). **c** Boxplot of SCZ PRS values for psychosis positive vs psychosis negative deletion carriers. **d** Distributions of score values for four SIPS variables displaying the strongest association with SCZ PRS (i.e., from left to right “disorganized communication” within the positive symptoms category, “social anhedonia” and “occupational functioning” within the negative symptoms category, and “impaired tolerance to normal stress” within the general symptoms category) over age and coloured by increasing SCZ PRS quintiles (dark blue, light blue, grey, orange, and red). Each dot represents a score determined at a given timepoint (visit) connected with straight line for each 22q11.2DS patient.

We first set out to determine whether the severity of clinical symptoms associated with psychosis can be explained by SCZ genetic load among 22q11.2 deletion carriers. To this end, we correlated SCZ PRS with 19 SIPS-derived items categorized into positive, negative, disorganized, and general symptoms. Cross-sectional analysis revealed that only “impaired tolerance to normal stress” was associated with SCZ PRS at FDR 5%, indicating that for one SD increase in SCZ PRS, the odds of scoring higher on the stress intolerance item doubled (OR 2.03, 95% CI 1.34–3.13, *P* = 0.001, Fig. 2a). When relaxing the FDR threshold to 10%, “social anhedonia” (OR 1.61, 95% CI 1.08–2.43, *P* = 0.02) and “ideational richness” (OR 1.69, 95% CI 1.14–2.54, *P* = 0.01) within negative symptoms, and “dysphoric mood” (OR 1.75, 95% CI 1.16–2.69, *P* = 0.009) within general symptoms, but none of the items within the positive symptoms category, showed a significant association with SCZ PRS (Fig. 2a; Supplementary Table 2, Supplementary Fig. 7). The distribution of SCZ PRS did not differ between psychosis positive and psychosis negative patients (*P* = 0.76, Fig. 2c).

To extend the findings of the cross-sectional analysis, we next investigated whether the 22q11.2DS patients with higher genomic burden for SCZ displayed steeper longitudinal increase/reduction on any symptomatic scale over time. To rule out false-positive associations due to small sample size, we used Bonferroni correction as well as bootstrapping validation. Random-effects ordinal regression modelling revealed that one SD increase in SCZ PRS corresponded on average to significantly greater odds to scoring higher on “disorganized communication” (OR 2.37, 95% CI 1.41–3.99) within positive symptoms, “social anhedonia” (OR 2.09, 95% CI 1.42–3.07), and “occupational functioning” (OR 1.82, 95% CI 1.32–2.51) within negative symptoms, “impairment in personal hygiene” (OR 1.82, 95% CI 1.29–2.56) within disorganized symptoms, and “dysphoric mood” (OR 2.0, 95% CI 1.28–3.11) and “impaired intolerance to normal stress” (OR 1.76, 95% CI 1.31–2.36) within general symptoms across time (Table 2, Fig. 2b, d; Supplementary Table 2, Supplementary Figs. 8, and 9). Whereas a sensitivity analysis did not allow to robustly show that SCZ PRS was correlated with negative and positive symptoms at different ages, we found in our longitudinal analysis with the younger sub-group (<18 years) that “disorganized communication” of positive symptoms showed stronger association with SCZ PRS, surviving Bonferroni correction (OR 2.95, 95% CI 1.43–6.30, *P* = 0.003), than “avolition” of negative symptoms that only survived FDR 10% correction (OR 1.55, 95% CI 1.05–2.29, *P* = 0.03; Supplementary Table 3, Supplementary Figs. 10 and 11). Altogether, our results suggest that 22q11.2DS patients with higher genetic liability to SCZ are specifically predisposed to a worsening negative and a general symptoms course.

**Table 2:** Longitudinal association analyses.

Ordinal variables				Continuous variables			
Positive symptoms	OR	95% CI	*P*	IQ variables	*β*	SE	*P*
Unusual Thought Content/ Delusional Ideas	1.78	1.16–2.73	0.008	Full scale IQ	−0.25	0.11	0.021*
Suspiciousness/Persecutory Ideas	1.57	1.07–2.29	0.02	verbal IQ	−0.25	0.11	0.024*
Grandiose Ideas	0.91	0.49–1.71	0.77	performance IQ	−0.19	0.11	0.077
Perceptual Abnormalities/ Hallucinations	1.34	0.86–2.09	0.20	**Brain volumes**	***β***	**SE**	***P***
Disorganized Communication	2.37	1.41–3.99	0.001*	Total cortical grey matter	−0.12	0.08	0.112
**Negative symptoms**	**OR**	**95% CI**	***P***	Right hippocampus	−0.28	0.10	0.0047*
Social Anhedonia	2.09	1.42–3.07	0.0002*	Left hippocampus	−0.23	0.10	0.0169*
Avolition	1.61	1.21–2.14	0.001	**Hippocampal subfields**	***β***	**SE**	***P***
Expression of Emotion	1.81	1.17–2.81	0.008	Left tail	−0.26	0.10	0.007
Experience of Emotions and Self	1.47	1.01–2.14	0.04	Left subiculum	−0.12	0.10	0.216
Ideational Richness	1.78	1.22–2.60	0.003	Left CA1	−0.14	0.10	0.132
Occupational Functioning	1.82	1.32–2.51	0.0003*	Left molecular layer	−0.12	0.10	0.235
**Disorganization symptoms**	**OR**	**95% CI**	***P***	Left GC-ML-DG	−0.09	0.10	0.344
Odd Behavior of Appearance	2.00	1.20–3.33	0.008	Left CA2/3	−0.13	0.10	0.186
Bizarre Thinking	1.93	1.08–3.42	0.025	Left CA4	−1.11	0.10	0.273
Trouble with Focus and Attention	1.71	1.12–2.60	0.013	Right tail	−0.18	0.10	0.088
Impairment in Personal Hygiene	1.82	1.29–2.56	0.0007*	Right subiculum	−0.18	0.10	0.079
**General symptoms**	**OR**	**95% CI**	***P***	Right CA1	−0.22	0.10	0.037
Sleep Disturbance	1.22	0.77–1.94	0.40	Right molecular layer	−0.17	0.10	0.084
Dysphoric Mood	2.00	1.28–3.11	0.002*	Right GC-ML-DG	−0.14	0.10	0.164
Motor Disturbances	1.64	1.05–2.55	0.028	Right CA2/3	−0.19	0.10	0.055
Impaired Tolerance to Normal Stress	1.76	1.31–2.36	0.0002*	Right CA4	−0.20	0.10	0.053

An asterisk indicates an association determined as significant after multiple testing.

*OR* odds ratio, *CI* confidence interval, *SE* standard error.

**Fig. 3:**
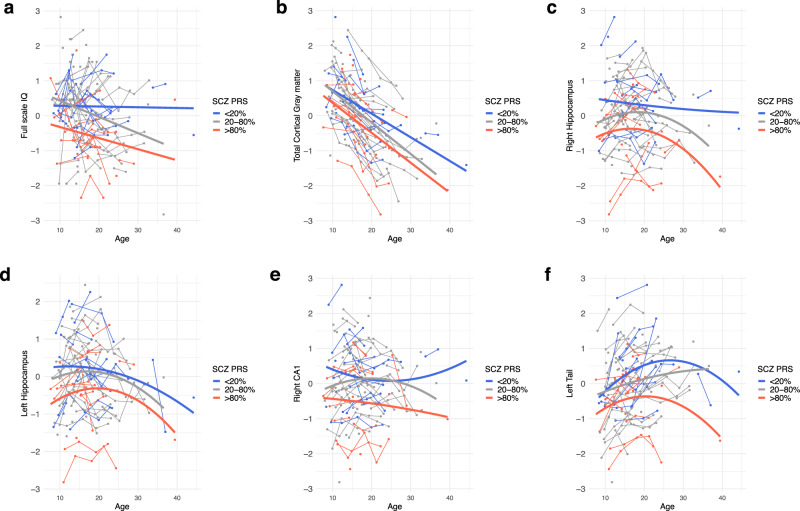
SCZ PRS association with cognition and brain imaging variables. Distribution of (**a**) FSIQ measurements and (**b**–**f**) volumetric MRI measurements (total cortical grey matter, right and left hippocampus, right CA1 and left tail) across time for 22q11.2DS patients. Each dot denotes a measurement determined at given timepoint (visit) connected by a straight line for each 22q11.2DS patient. The subjects are coloured based on their clustering on SCZ PRS distribution. The blue and red denote the lowest and the highest SCZ PRS quintile, respectively, with grey marking joint three middle quintiles.

We next interrogated whether higher genetic burden for SCZ predisposes 22q11.2DS patients to a worsening in the trajectory of cognitive abilities. While none of the IQ variables reached statistical significance threshold in cross-sectional analysis (Supplementary Table 4), mixed linear modelling using longitudinal FSIQ measurements revealed a significant association between increasing SCZ PRS and cognitive decline (*β* = –0.25, standard error (SE) 0.11, *P* = 0.02, Table 2, Fig. 3a; Supplementary Table 4). It was driven by more severe decline in verbal capabilities (VIQ, *β* = –0.25, SE 0.11, *P* = 0.02), rather than underperformance in visuospatial intellectual abilities (PIQ, *β* = –0.19, SE 0.1, *P* = 0.08; Table 2; Supplementary Table 4, Supplementary Fig. 12) with one SD increase in PRS predicting a 3-point lower VIQ level on average.

Lastly and given previous findings linking SCZ PRS with cortical and hippocampal features in the general population [30–32] and 22q11.2DS patients displaying significantly increased variability in hippocampal volume compared to control subjects [75], we set out to investigate whether SCZ PRS contributes to volumetric reduction of hippocampus and total cortical grey matter among 22q11.2DS patients. While SCZ PRS was not associated with volumetric reduction in total cortical grey matter cross-sectionally nor longitudinally (Table 2, Fig. 3b; Supplementary Table 5), the volumes of both right and left hippocampus showed significant reduction upon increase in SCZ genetic load in both cross-sectional (*β* = –0.30, SE = 0.10, *P* = 0.004; *β* = –0.28, SE = 0.10, *P* = 0.01, respectively) and longitudinal analysis (*β* = –0.28, SE = 0.10, *P* = 0.005; *β* = –0.28, SE = 0.10, *P* = 0.017, respectively; Table 2, Fig. 3c, d; Supplementary Table 5). While a “region of interest” analysis for hippocampal subfields revealed a more pronounced signal in longitudinal analysis; namely, that higher SCZ PRS was associated with the longitudinal volumetric reduction of left tail (*β* = –0.26, SE = 0.10, *P* = 0.007), and right CA1 region (*β* = –0.22, SE = 0.10, *P* = 0.037; Table 2, Fig. 3e, f; Supplementary Table 5) at nominal significance, these did not survive multiple correction.

## Discussion

The high but incomplete penetrance of clinical manifestations among 22q11.2 deletion carriers likely result from the interplay between multiple molecular mechanisms. Given the highly polygenic nature of SCZ and the convergence of its genetic liability with biologically overlapping outcomes, we assessed whether SCZ PRS contributes to a worsened endophenotypic course among patients harbouring such a large-effect genetic variant. We ascertained that the polygenic contribution to SCZ acts upon the threshold-lowering first hit (i.e., the deletion) in modifying the endophenotypes of 22q11.2DS. It augments the derailment of the developmental trajectories for psychosis-risk symptoms, cognition, and hippocampal volume. While it remains to be investigated how the genetically predisposed molecular deviations captured in a PRS explicitly result in 22q11.2DS manifestation, our findings suggest the following implications.

Firstly, we identified that the higher polygenic burden for SCZ was chiefly associated with the negative and the general rather than with the positive symptoms course, contradicting previous findings identified for 22q11.2 deletion carriers [20, 21], yet corroborating results obtained in the general population [25–27]. It was hypothesized that the genetic liability for SCZ might more strongly index molecular pathways manifesting as negative and general symptoms which in essence can reflect broad and heterogeneous clinical outcomes, and only weakly affect mechanisms that result in positive symptoms such as hallucinations and delusions [26]. Additionally, the diminished gene dosage resulting from the 22q11.2 deletion per se might account for the development of positive symptoms through mechanisms not captured by PRS [76]. However, but not contradictorily, given that the sample sets assessed in previous 22q11.2DS studies were considerably older [20, 21], and that the longitudinal analysis for symptoms course in the current study did indicate a positive association between SCZ PRS and delusional and persecutory ideas at more relaxed multiple test correction (Table 2; Supplementary Table 2), it is possible that patients at higher polygenic risk are yet to develop psychosis to its full extent. While our study with its low sample size and age range does not properly allow to assess whether SCZ PRS correlates with different symptom dimensions at different ages, our preliminary results warrant further investigation.

Secondly, as expected, the polygenic burden for SCZ amplified cognitive decline among 22q11.2DS patients. This result recapitulates the negative genetic correlation between cognition and SCZ [77–79] as well as replicates the previous report for 22q11.2DS patients [20]. It remains to be investigated whether the 22q11.2DS patients at increased genetic risk for SCZ and with lower cognitive levels exhibit more severe psychosis transitions compared to those with low SCZ PRS, and whether the stronger association with verbal IQ results from common variant burden functioning through domains affecting verbal rather than visuospatial abilities. In support with this hypothesis, 22q11.2 patients with psychotic symptoms did show an earlier decline specifically in VIQ [7]. Nevertheless, given that the higher levels of negative symptoms combined with the lower levels of cognition precede psychosis development [25, 80–82] and that the effect of the polygenic burden on SCZ could be partially mediated through cognition-relevant pathways [24], our results support the neurodevelopmental continuum model for psychosis [83]. These also indicate that the assessment of SCZ polygenic burden could provide valuable information for prognosis, patient monitoring and treatment allocation for 22q11.2DS patients.

Thirdly, the association between SCZ PRS and bilateral hippocampal volume reduction points out that the reduced hippocampal volume present in 22q11.2 patients [52, 84–86] is further aggravated by SCZ genome-wide burden. Prior estimates displaying a genetic overlap between idiopathic SCZ and hippocampal volume [87, 88] support the hypothesis that deviations from the normal hippocampal developmental trajectory could be a genetically-mediated intermediate phenotype for SCZ risk [32]. While we did not replicate the prior published association between SCZ PRS and left CA2/3 [31], we identified a more pronounced effect of SCZ PRS on the right CA1 and the left tail region, although these associations did not survive multiple testing. Interestingly, the hippocampal tail is enriched in expression of SCZ-related genes [88]. Our inverse association between hippocampal volume and polygenic burden for SCZ substantiates the hypothesis that hippocampus plays a central role in SCZ pathophysiology [89].

We are fully aware of the limitations of the present study starting by its modest sample size. Our estimates display large confidence intervals that should be interpreted with caution. We could not compare the endophenotypic trajectories with those measured in healthy controls as the latter group consists of first-degree siblings in the Swiss 22q11.2 cohort. Nevertheless, the accrued comprehensive brain imaging and clinical measurements of deletion carriers over an extensive time period provides a unique dataset for association testing and for delineating longitudinal phenotype-specific trajectories rather than cross-sectional snapshot associations, while being at the same time sub-optimal for replication studies. To increase power in capturing the genetic liability for SCZ, we considered the latest wave of SCZ GWAS that utilized the largest case-control dataset to date [22] as well as applied a PRS calculation method shown to outperform methods used in previous studies [20, 21, 25], thereby potentially resulting in more accurate downstream assessment with trait associated variables [90]. By using an external ancestrally matched cohort for deriving and validating the best performing SCZ, we recapitulated prior assessments substantiating that SCZ is highly polygenic with genetic effects diluted across the whole genome [22, 72, 73]. We acknowledge that a Swiss population-specific dataset would have allowed to derive the optimal SCZ PRS for association testing, but such data are unavailable. To minimize any bias stemming from sub-population stratification, we limited SCZ PRS calculation in the EstBB and in the Swiss 22q11.2DS cohort to a strictly common set of SNVs and used only samples of European ancestry that match the genetic background of samples used in SCZ GWAS [22]. No discordance in SCZ PRS value distributions was identified between the two datasets (Supplementary Fig. 6b). Furthermore, given that transferring a PRS to a different population but with the same ancestral background results in underestimation rather than in overestimation of risk prediction [91], the associations identified in the current study could be considered as conservative estimates. Lastly, given the small sample size and multiple testing burden, we could not reasonably perform a discovery analysis to identify brain regions most significantly impacted by SCZ polygenic burden but had to restrict ourselves to a candidate approach. Still, our results for all cortical and sub-cortical volume, surface area and thickness measurements according to the Desikan Killiany atlas indicate that hippocampus exhibits the strongest signal and is in line with previous reports [30, 31, 84, 89] (Supplementary Table 7).

In conclusion, our findings support the notion that the phenotypic expression resulting from a large-effect genetic variant is modified by second lower-effect SNVs. We demonstrate here that the higher polygenic burden for SCZ is associated with a worsened symptoms course, cognitive decline, and hippocampal volume reduction in 22q11.2 deletion carriers. These results substantiate that a genome-wide integrative analysis of allelic variation across the entire frequency spectrum is required to fully comprehend the genetic architecture and phenotypic variability of developmental disorders caused by a high-effect genetic variant [12, 19, 92–94]. Whether large-effect variants and polygenic burden act independently and additively or operate epistatically warrants investigation.

## Supplementary information

Supplementary Information

## References

[1] Henrichsen CN, Chaignat E, Reymond A (2009). Copy number variants, diseases and gene expression. Hum Mol Genet.

[2] Olsen L, Sparso T, Weinsheimer SM, Dos Santos MBQ, Mazin W, Rosengren A (2018). Prevalence of rearrangements in the 22q11.2 region and population-based risk of neuropsychiatric and developmental disorders in a Danish population: a case-cohort study. Lancet Psychiatry.

[3] D’Angelo D, Lebon S, Chen Q, Martin-Brevet S, Snyder LG, Hippolyte L (2016). Defining the effect of the 16p11.2 duplication on cognition, behavior, and medical comorbidities. JAMA Psychiatry.

[4] Mannik K, Magi R, Mace A, Cole B, Guyatt AL, Shihab HA (2015). Copy number variations and cognitive phenotypes in unselected populations. JAMA.

[5] Zufferey F, Sherr EH, Beckmann ND, Hanson E, Maillard AM, Hippolyte L (2012). A 600 kb deletion syndrome at 16p11.2 leads to energy imbalance and neuropsychiatric disorders. J Med Genet.

[6] McDonald-McGinn DM, Sullivan KE, Marino B, Philip N, Swillen A, Vorstman JAS (2015). 22q11.2 deletion syndrome. Nat Rev Dis Prim.

[7] Vorstman JAS, Breetvelt EJ, Duijff SN, Eliez S, Schneider M, Jalbrzikowski M (2015). Cognitive decline preceding the onset of psychosis in patients with 22q11.2 deletion syndrome. JAMA Psychiatry.

[8] Kim EH, Yum MS, Lee BH, Kim HW, Lee HJ, Kim GH (2016). Epilepsy and other neuropsychiatric manifestations in children and adolescents with 22q11.2 deletion syndrome. J Clin Neurol.

[9] Jonas RK, Montojo CA, Bearden CE (2014). The 22q11.2 deletion syndrome as a window into complex neuropsychiatric disorders over the lifespan. Biol Psychiatry.

[10] Schneider M, Debbane M, Bassett AS, Chow EW, Fung WL, van den Bree M (2014). Psychiatric disorders from childhood to adulthood in 22q11.2 deletion syndrome: results from the International Consortium on Brain and Behavior in 22q11.2 Deletion Syndrome. Am J Psychiatry.

[11] Murphy KC, Jones LA, Owen MJ (1999). High rates of schizophrenia in adults with velo-cardio-facial syndrome. Arch Gen Psychiatry.

[12] Pizzo L, Jensen M, Polyak A, Rosenfeld JA, Mannik K, Krishnan A (2019). Rare variants in the genetic background modulate cognitive and developmental phenotypes in individuals carrying disease-associated variants. Genet Med.

[13] Fiksinski AM, Schneider M, Zinkstok J, Baribeau D, Chawner S, Vorstman JAS (2021). Neurodevelopmental trajectories and psychiatric morbidity: lessons learned from the 22q11.2 deletion syndrome. Curr Psychiatry Rep..

[14] Zinkstok JR, Boot E, Bassett AS, Hiroi N, Butcher NJ, Vingerhoets C (2019). Neurobiological perspective of 22q11.2 deletion syndrome. Lancet Psychiatry.

[15] Ching CRK, Gutman BA, Sun D, Villalon Reina J, Ragothaman A, Isaev D (2020). Mapping subcortical brain alterations in 22q11.2 deletion syndrome: effects of deletion size and convergence with idiopathic neuropsychiatric illness. Am J Psychiatry.

[16] Merico D, Zarrei M, Costain G, Ogura L, Alipanahi B, Gazzellone MJ (2015). Whole-genome sequencing suggests Schizophrenia risk mechanisms in humans with 22q11.2 deletion syndrome. G3.

[17] Morrow BE, McDonald-McGinn DM, Emanuel BS, Vermeesch JR, Scambler PJ (2018). Molecular genetics of 22q11.2 deletion syndrome. Am J Med Genet A.

[18] Bassett AS, Lowther C, Merico D, Costain G, Chow EWC, van Amelsvoort T (2017). Rare genome-wide copy number variation and expression of Schizophrenia in 22q11.2 deletion syndrome. Am J Psychiatry.

[19] Tansey KE, Rees E, Linden DE, Ripke S, Chambert KD, Moran JL (2016). Common alleles contribute to schizophrenia in CNV carriers. Mol Psychiatry.

[20] Davies RW, Fiksinski AM, Breetvelt EJ, Williams NM, Hooper SR, Monfeuga T (2020). Using common genetic variation to examine phenotypic expression and risk prediction in 22q11.2 deletion syndrome. Nat Med.

[21] Cleynen I, Engchuan W, Hestand MS, Heung T, Holleman AM, Johnston HR (2021). Genetic contributors to risk of schizophrenia in the presence of a 22q11.2 deletion. Mol Psychiatry.

[22] Trubetskoy V, Pardinas AF, Qi T, Panagiotaropoulou G, Awasthi S, Bigdeli TB (2022). Mapping genomic loci implicates genes and synaptic biology in schizophrenia. Nature.

[23] Serdarevic F, Jansen PR, Ghassabian A, White T, Jaddoe VWV, Posthuma D (2018). Association of genetic risk for Schizophrenia and bipolar disorder with infant neuromotor development. JAMA Psychiatry.

[24] Toulopoulou T, Zhang XW, Cherny S, Dickinson D, Berman KF, Straub RE (2019). Polygenic risk score increases schizophrenia liability through cognition-relevant pathways. Brain.

[25] Legge SE, Cardno AG, Allardyce J, Dennison C, Hubbard L, Pardinas AF (2021). Associations between Schizophrenia polygenic liability, symptom dimensions, and cognitive ability in Schizophrenia. JAMA Psychiatry.

[26] Jones HJ, Stergiakouli E, Tansey KE, Hubbard L, Heron J, Cannon M (2016). Phenotypic manifestation of genetic risk for Schizophrenia during adolescence in the general population. JAMA Psychiatry.

[27] Jonas KG, Lencz T, Li K, Malhotra AK, Perlman G, Fochtmann LJ (2019). Schizophrenia polygenic risk score and 20-year course of illness in psychotic disorders. Transl Psychiatry.

[28] van Scheltinga AFT, Bakker SC, van Haren NEM, Derks EM, Buizer-Voskamp JE, Boos HBM (2013). Genetic Schizophrenia risk variants jointly modulate total brain and white matter volume. Biol Psychiatry.

[29] Neilson E, Shen XY, Cox SR, Clarke TK, Wigmore EM, Gibson J (2019). Impact of polygenic risk for Schizophrenia on cortical structure in UK biobank. Biol Psychiatry.

[30] Stauffer EM, Bethlehem RAI, Warrier V, Murray GK, Romero-Garcia R, Seidlitz J (2021). Grey and white matter microstructure is associated with polygenic risk for schizophrenia. Mol Psychiatry.

[31] Alnaes D, Kaufmann T, van der Meer D (2019). Brain heterogeneity in Schizophrenia and its association with polygenic risk. Jama Psychiatry.

[32] Chen Q, Ursini G, Romer AL, Knodt AR, Mezeivtch K, Xiao E (2018). Schizophrenia polygenic risk score predicts mnemonic hippocampal activity. Brain.

[33] Schaer M, Debbane M, Bach Cuadra M, Ottet MC, Glaser B, Thiran JP (2009). Deviant trajectories of cortical maturation in 22q11.2 deletion syndrome (22q11DS): a cross-sectional and longitudinal study. Schizophr Res.

[34] Shaikh TH, Kurahashi H, Emanuel BS (2001). Evolutionarily conserved low copy repeats (LCRs) in 22q11 mediate deletions, duplications, translocations, and genomic instability: An update and literature review. Genet Med.

[35] Edelmann L, Pandita RK, Morrow BE (1999). Low-copy repeats mediate the common 3-Mb deletion in patients with velo-cardio-facial syndrome. Am J Hum Genet.

[36] Shaikh TH, Kurahashi H, Saitta SC, O’Hare AM, Hu P, Roe BA (2000). Chromosome 22-specific low copy repeats and the 22q11.2 deletion syndrome: genomic organization and deletion endpoint analysis. Hum Mol Genet.

[37] Saitta SC, Harris SE, Gaeth AP, Driscoll DA, McDonald-McGinn DM, Maisenbacher MK (2004). Aberrant interchromosomal exchanges are the predominant cause of the 22q11.2 deletion. Hum Mol Genet.

[38] Carlson C, Sirotkin H, Pandita R, Goldberg R, McKie J, Wadey R (1997). Molecular definition of 22q11 deletions in 151 velo-cardio-facial syndrome patients. Am J Hum Genet.

[39] Miller TJ, McGlashan TH, Rosen JL, Cadenhead K, Cannon T, Ventura J (2003). Prodromal assessment with the structured interview for prodromal syndromes and the scale of prodromal symptoms: predictive validity, interrater reliability, and training to reliability. Schizophr Bull.

[40] Weisman O, Guri Y, Gur RE, McDonald-McGinn DM, Calkins ME, Tang SX (2017). Subthreshold psychosis in 22q11.2 deletion syndrome: multisite naturalistic study. Schizophr Bull.

[41] Tang SX, Yi JJ, Moore TM, Calkins ME, Kohler CG, Whinna DA (2014). Subthreshold psychotic symptoms in 22q11.2 deletion syndrome. J Am Acad Child Psy.

[42] Miller TJ, McGlashan TH, Rosen JL, Somjee L, Markovich PJ, Stein K (2002). Prospective diagnosis of the initial prodrome for schizophrenia based on the structured interview for prodromal syndromes: Preliminary evidence of interrater reliability and predictive validity. Am J Psychiatry.

[43] Wechsler D Wechsler Adult Intelligence Scale-IV: administration and scoring manual. *Psychological Corporation*. 2011.

[44] Wechsler D The Wechsler intelligence scale for children, 4th editon. *Pearson Assessment*. 2004.

[45] Mancini V, Maeder J, Bortolin K, Schneider M, Schaer M, Eliez S (2021). Long-term effects of early treatment with SSRIs on cognition and brain development in individuals with 22q11.2 deletion syndrome. Transl Psychiatry.

[46] Maeder J, Schneider M, Bostelmann M, Debbane M, Glaser B, Menghetti S (2016). Developmental trajectories of executive functions in 22q11.2 deletion syndrome. J Neurodev Disord.

[47] Fischl B, Salat DH, Busa E, Albert M, Dieterich M, Haselgrove C (2002). Whole brain segmentation: Automated labeling of neuroanatomical structures in the human brain. Neuron.

[48] Fischl B, Dale AM (2000). Measuring the thickness of the human cerebral cortex from magnetic resonance images. Proc Natl Acad Sci USA.

[49] Fischl B, Sereno MI, Dale AM (1999). Cortical surface-based analysis. II: Inflation, flattening, and a surface-based coordinate system. Neuroimage.

[50] Desikan RS, Segonne F, Fischl B, Quinn BT, Dickerson BC, Blacker D (2006). An automated labeling system for subdividing the human cerebral cortex on MRI scans into gyral based regions of interest. Neuroimage.

[51] Iglesias JE, Augustinack JC, Nguyen K, Player CM, Player A, Wright M (2015). A computational atlas of the hippocampal formation using ex vivo, ultra-high resolution MRI: application to adaptive segmentation of in vivo MRI. Neuroimage.

[52] Mancini V, Sandini C, Padula MC, Zoller D, Schneider M, Schaer M (2020). Positive psychotic symptoms are associated with divergent developmental trajectories of hippocampal volume during late adolescence in patients with 22q11DS. Mol Psychiatry.

[53] Chang CC, Chow CC, Tellier LC, Vattikuti S, Purcell SM, Lee JJ (2015). Second-generation PLINK: rising to the challenge of larger and richer datasets. Gigascience.

[54] Danecek P, McCarthy SA, Durbin R (2016). HipSci consortium. a method for checking genomic integrity in cultured cell lines from SNP genotyping data. Plos One.

[55] Altshuler DM, Durbin RM, Abecasis GR, Bentley DR, Chakravarti A, Clark AG (2015). A global reference for human genetic variation. Nature.

[56] Delaneau O, Ongen H, Brown AA, Fort A, Panousis NI, Dermitzakis ET (2017). A complete tool set for molecular QTL discovery and analysis. Nat Commun.

[57] McCarthy S, Das S, Kretzschmar W, Delaneau O, Wood AR, Teumer A (2016). A reference panel of 64,976 haplotypes for genotype imputation. Nat Genet.

[58] Leitsalu L, Haller T, Esko T, Tammesoo ML, Alavere H, Snieder H (2015). Cohort Profile: Estonian Biobank of the Estonian Genome Center, University of Tartu. Int J Epidemiol.

[59] Vilhjalmsson BJ, Yang J, Finucane HK, Gusev A, Lindstrom S, Ripke S (2015). Modeling linkage disequilibrium increases accuracy of polygenic risk scores. Am J Hum Genet.

[60] Loh PR, Danecek P, Palamara PF, Fuchsberger C, Reshef YA, Finucane HK (2016). Reference-based phasing using the Haplotype Reference Consortium panel. Nat Genet.

[61] Browning SR, Browning BL (2007). Rapid and accurate haplotype phasing and missing-data inference for whole-genome association studies by use of localized haplotype clustering. Am J Hum Genet.

[62] Mitt M, Kals M, Parn K (2017). Improved imputation accuracy of rare and low-frequency genetic variants using population-specific high-coverage whole-genome sequencing data based imputation reference panel. Eur J Hum Genet.

[63] Brainstorm C, Anttila V, Bulik-Sullivan B, Finucane HK, Walters RK, Bras J (2018). Analysis of shared heritability in common disorders of the brain. Science.

[64] Robin X, Turck N, Hainard A, Tiberti N, Lisacek F, Sanchez JC (2011). pROC: an open-source package for R and S plus to analyze and compare ROC curves. BMC Bioinforma.

[65] Therneau TM A Package for Survival Analysis in R. *R package version* 3.2-13 2021. https://CRAN.R-project.org/package=survival.

[66] Venables WN, Ripley BD Modern Applied Statistics with S, 4th Edition. Springer, New York 2002. https://www.stats.ox.ac.uk/pub/MASS4.

[67] Christensen RHB ordinal---Regression Models for Ordinal Data. *R package version* 2019*;* https://CRAN.R-project.org/package=ordinal.

[68] Brant R (1990). Assessing proportionality in the proportional odds model for ordinal logistic-regression. Biometrics.

[69] Storey JD, Tibshirani R (2003). Statistical significance for genomewide studies. Proc Natl Acad Sci USA.

[70] Bates D, Machler M, Bolker BM, Walker SC (2015). Fitting linear mixed-effects models using lme4. J Stat Softw.

[71] R: A Language and Environment for Statistical Computing. R Foundation for Statistical Computing 2017.

[72] Schizophrenia Working Group of the Psychiatric Genomics C. (2014). Biological insights from 108 schizophrenia-associated genetic loci. Nature.

[73] Ni G, Zeng J, Revez JA, Wang Y, Zheng Z, Ge T (2021). A comparison of ten polygenic score methods for psychiatric disorders applied across multiple cohorts. Biol Psychiatry.

[74] Calafato MS, Thygesen JH, Ranlund S, Zartaloudi E, Cahn W, Crespo-Facorro B (2018). Use of schizophrenia and bipolar disorder polygenic risk scores to identify psychotic disorders. Br J Psychiatry.

[75] Rogdaki M, Gudbrandsen M, McCutcheon RA, Blackmore CE, Brugger S, Ecker C (2020). Magnitude and heterogeneity of brain structural abnormalities in 22q11.2 deletion syndrome: a meta-analysis. Mol Psychiatry.

[76] Bergen SE, Ploner A, Howrigan D, O’Donovan MC, Group CNVA, the Schizophrenia Working Group of the Psychiatric Genomics C (2019). Joint Contributions of Rare Copy Number Variants and Common SNPs to Risk for Schizophrenia. Am J Psychiatry.

[77] Savage JE, Jansen PR, Stringer S, Watanabe K, Bryois J, de Leeuw CA (2018). Genome-wide association meta-analysis in 269,867 individuals identifies new genetic and functional links to intelligence. Nat Genet.

[78] Ohi K, Sumiyoshi C, Fujino H, Yasuda Y, Yamamori H, Fujimoto M (2018). Genetic Overlap between General Cognitive Function and Schizophrenia: A Review of Cognitive GWASs. Int J Mol Sci.

[79] Davies G, Lam M, Harris SE, Trampush JW, Luciano M, Hill WD (2019). Study of 300,486 individuals identifies 148 independent genetic loci influencing general cognitive function. Nat Commun.

[80] Schneider M, Armando M, Schultze-Lutter F, Pontillo M, Vicari S, Debbane M (2019). Prevalence, course and psychosis-predictive value of negative symptoms in 22q11.2 deletion syndrome. Schizophr Res.

[81] Hemager N, Plessen KJ, Thorup A, Christiani C, Ellersgaard D, Spang KS (2018). Assessment of Neurocognitive Functions in 7-Year-Old Children at Familial High Risk for Schizophrenia or Bipolar Disorder The Danish High Risk and Resilience Study VIA 7. Jama Psychiatry.

[82] Schneider M, Van der Linden M, Menghetti S, Glaser B, Debbane M, Eliez S (2014). Predominant negative symptoms in 22q11.2 deletion syndrome and their associations with cognitive functioning and functional outcome. J Psychiatr Res.

[83] Owen MJ, O’Donovan MC (2017). Schizophrenia and the neurodevelopmental continuum: evidence from genomics. World Psychiatry.

[84] Flahault A, Schaer M, Ottet MC, Debbane M, Eliez S (2012). Hippocampal volume reduction in chromosome 22q11.2 deletion syndrome (22q11.2DS): A longitudinal study of morphometry and symptomatology. Psychiatry Res.

[85] Delavari F, Sandini C, Zoller D, Mancini V, Bortolin K, Schneider M (2021). Dysmaturation observed as altered hippocampal functional connectivity at rest is associated with the emergence of positive psychotic symptoms in patients with 22q11 deletion syndrome. Biol Psychiatry.

[86] Debbane M, Schaer M, Farhoumand R, Glaser B, Eliez S (2006). Hippocampal volume reduction in 22q11.2 deletion syndrome. Neuropsychologia.

[87] Smeland OB, Wang YP, Frei O, Li W, Hibar DP, Franke B (2018). Genetic overlap between Schizophrenia and volumes of hippocampus, putamen, and intracranial volume indicates shared molecular genetic mechanisms. Schizophr Bull.

[88] van der Meer D, Rokicki J, Kaufmann T, Cordova-Palomera A, Moberget T, Alnaes D (2020). Brain scans from 21,297 individuals reveal the genetic architecture of hippocampal subfield volumes. Mol Psychiatry.

[89] Lieberman JA, Girgis RR, Brucato G, Moore H, Provenzano F, Kegeles L (2018). Hippocampal dysfunction in the pathophysiology of schizophrenia: a selective review and hypothesis for early detection and intervention. Mol Psychiatry.

[90] Khera AV, Chaffin M, Aragam KG, Haas ME, Roselli C, Choi SH (2018). Genome-wide polygenic scores for common diseases identify individuals with risk equivalent to monogenic mutations. Nat Genet.

[91] Gola D, Erdmann J, Lall K, Magi R, Muller-Myhsok B, Schunkert H (2020). Population bias in polygenic risk prediction models for coronary artery disease. Circ Genom Precis Med.

[92] Katsanis N (2016). The continuum of causality in human genetic disorders. Genome Biol.

[93] Niemi MEK, Martin HC, Rice DL, Gallone G, Gordon S, Kelemen M (2018). Common genetic variants contribute to risk of rare severe neurodevelopmental disorders. Nature.

[94] Oetjens MT, Kelly MA, Sturm AC, Martin CL, Ledbetter DH (2019). Quantifying the polygenic contribution to variable expressivity in eleven rare genetic disorders. Nat Commun.

