# Supplementary material for: Contribution of schizophrenia polygenic burden to longitudinal phenotypic variance in 22q11.2 deletion syndrome
Source: Mol Psychiatry. 2022 Jun 29;27(10):4191–200. doi: 10.1038/s41380-022-01674-9 (PMC9718680; doi:10.1038/s41380-022-01674-9)
Supplement: Supplementary file 1 — Supplementary Information [file 41380_2022_1674_MOESM1_ESM.pdf]

Supplementary Information

Contribution of schizophrenia polygenic burden to longitudinal phenotypic variance in 22q11.2 deletion syndrome

Maris Alver PhD[1,2,3], Valentina Mancini MD[4], Kristi Läll PhD[2], Maude Schneider PhD[4,5], Luciana Romano[1], Estonian Biobank Research Team[2], Reedik Mägi PhD[2], Emmanouil T. Dermitzakis PhD[1], Stephan Eliez MD PhD[1,4] & Alexandre Reymond PhD[3]

[1] Department of Genetic Medicine and Development, University of Geneva School of Medicine, Geneva, Switzerland
[2] Estonian Genome Center, Institute of Genomics, University of Tartu, Tartu, Estonia
[3] Center for Integrative Genomics, University of Lausanne, Lausanne, Switzerland
[4] Developmental Imaging and Psychopathology Laboratory, University of Geneva School of Medicine, Geneva, Switzerland
[5] Clinical Psychology Unit for Intellectual and Developmental Disabilities, Faculty of Psychology and Educational Sciences, University of Geneva, Switzerland

This document includes:
Supplementary Note
Supplementary Figures 1 to 12
Supplementary Tables 1 to 7

Supplementary Note

Overview of schizophrenia (SCZ) case selection in EstBB

The Estonian Biobank acquires phenotype data through annual linking to electronic health repositories. These include diagnoses reported by clinical physicians in the two main hospitals (North-Estonia Medical Center and Tartu University Hospital), eHealth (nationwide system integrating data from different healthcare providers in Estonia for establishing a common record for each individual), Population Registry, Cancer Registry, Causes of Death Registry, Myocardial Infarction Registry and Tuberculosis Registry with the biggest proportion (>80% of the diagnoses) retrieved from the Estonian Health Insurance Fund (EHIF)[1]. EHIF is an administrative database that pools detailed and person-level billing data on all health care services provided to all insured individuals in Estonia from year 2000 (i.e., information on all established diagnoses). EHIF is the only organization in Estonia handling compulsory health insurance that covers 95.5% of the population. EHIF is mandated by law to examine the quality and necessity of provided services and accuracy of the documentation (Estonian Health Insurance Fund Act, 2000)[2]. To this end, approximately 0.1% of all issued bills are manually checked by physicians at EHIF with annual reports retrievable in Estonian[3]. Data are at individual level and linkage to the Estonian Biobank cohort is conducted via the unique personal identification number. Given that the public share of health care is about 80% and private financing of health care is mainly related to dental care and pharmaceuticals, EHIF database contains information virtually about the entire health care provided in Estonia. Additionally, in 2015, the World Bank Group investigated the health care integration in Estonia. With the data taken solely from the EHIF database, it was determined as an adequate data source representative of the whole Estonian health care service[4,5].

In this study, SCZ cases were identified if an EstBB participant carried at least one report of an ICD-10 code for Schizophrenia Spectrum Disorder (F2*) as the main diagnosis given by a psychiatrist or a neurologist (i.e., excluded those carrying SCZ diagnosis as a comorbid condition or were diagnosed by a non-specialist). The ICD-10 F2* sub-codes are as follows: F20 – Schizophrenia; F21 – Schizotypical disorder; F22 – Delusional disorders; F23 – Brief psychotic disorder; F24 – Shared psychotic disorder; F25 – Schizoaffective disorders; F28 – Other psychotic disorder not due to substance or known physiological condition; F29 – Unspecified psychosis not due to a substance or known psychological condition. Controls were ascertained if no such diagnosis was reported. In all sets, cases with bipolar disorder and/or mania (ICD-10 F30.*, F31.*) were excluded given the considerable genetic overlap between these distinct psychiatric disorders and SCZ[6]. Mis- or underdiagnosis of SCZ and misreporting of the diagnosis in electronic health records is mitigated by using a large set of control samples. We do not expect SCZ diagnosis, a severe debilitating disorder, to be given lightly to acquire a treatment bill nor a medication prescription as there is no pharmaceutical treatment for SCZ that does not cause considerable side effects.

References

1. Leitsalu L, Haller T, Esko T, Tammesoo ML, Alavere H, Snieder H, et al. Cohort Profile: Estonian Biobank of the Estonian Genome Center, University of Tartu. *Int J Epidemiol* 2015; **44**: 1137-47.
2. Estonian Health Insurance Fund Act: https://www.riigiteataja.ee/en/compare_original/530102013071
3. Annual reports of EHIF: https://www.haigekassa.ee/en/annual-reports
4. The State of Health Care Integration in Estonia Summary Report by the World Bank Group: https://www.haigekassa.ee/sites/default/files/Maailmapanga-uuring/veeb_eng_summary_report_hk_2015_mai.pdf
5. Saar A, Läll K, Alver M, Marandi T, Ainla T, Eha J, Metspalu A, Fischer K. Estimating the performance of three cardiovascular disease risk scores: the Estonian Biobank cohort study. *J Epidemiol Community Health* 2019; **73**: 272-277.
6. Brainstorm Consortium, Anttila V, Bulik-Sullivan B, Finucane HK, Walters RK, Bras J, et al. Analysis of shared heritability in common disorders of the brain. *Science* 2018; **360**: eaap8757.

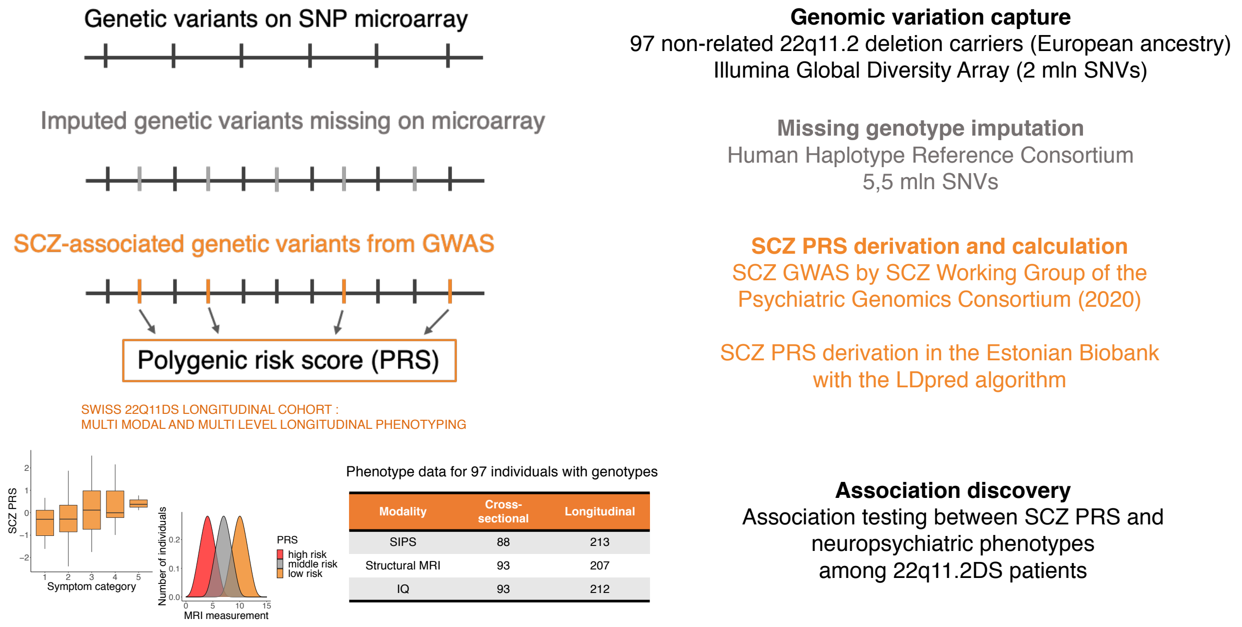

Supplementary Figure 1. Overview of the study design and Swiss 22q11.2DS cohort data used for association testing.

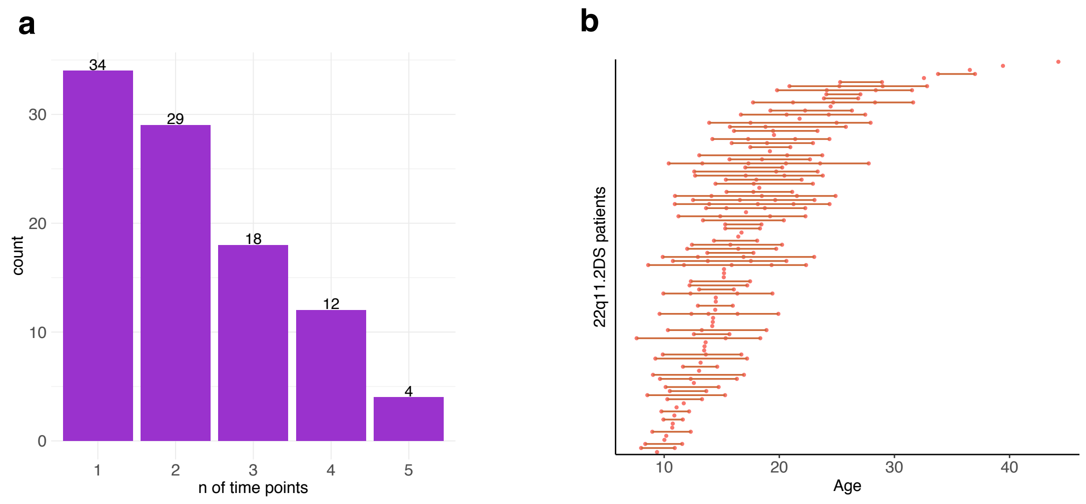

Supplementary Figure 2. Overview of the phenotypic characteristics within the Swiss 22q11.2DS cohort. Overview is given for the 97 individuals who passed genotype quality control and had phenotype data available. (a) Count of individuals with phenotypic data acquired at multiple timepoints. (b) Distribution of age at timepoint visits for 22q11.2DS patients with visits outlined with a dot and connected with a straight line for each subject.

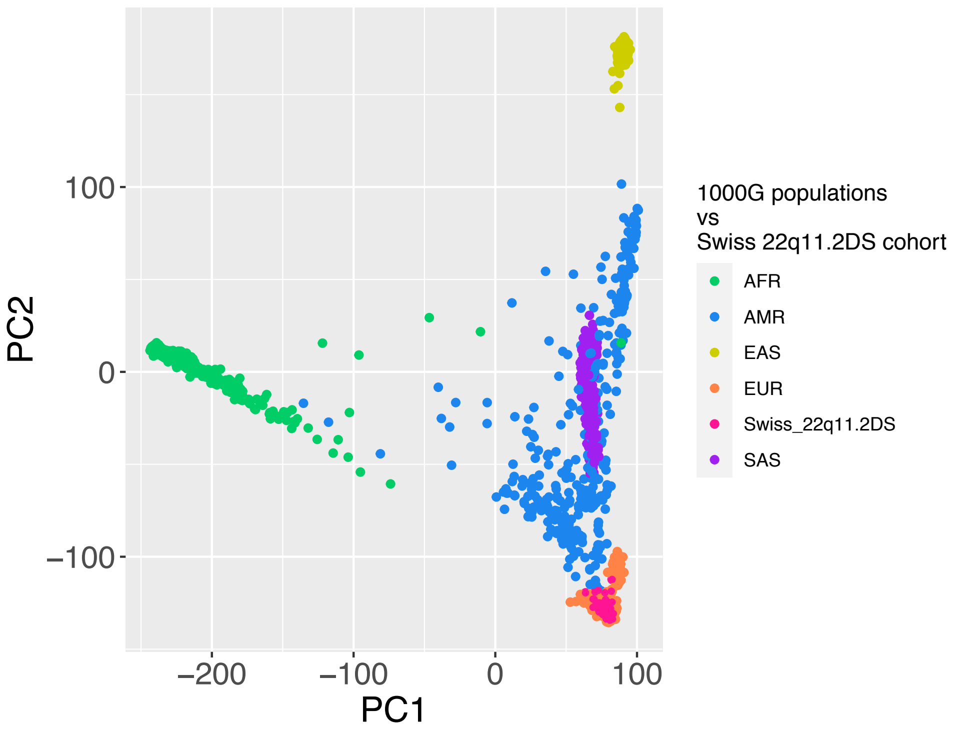

Supplementary Figure 3. Principal component (PC) analysis of genotype data. Coordinates of PC1 vs PC2 in reference to the 1000 Genome Project samples. The Swiss 22q11.2DS cohort samples are coloured in pink and cluster at PC coordinates represented by the European super population in the 1000 Genome Project.

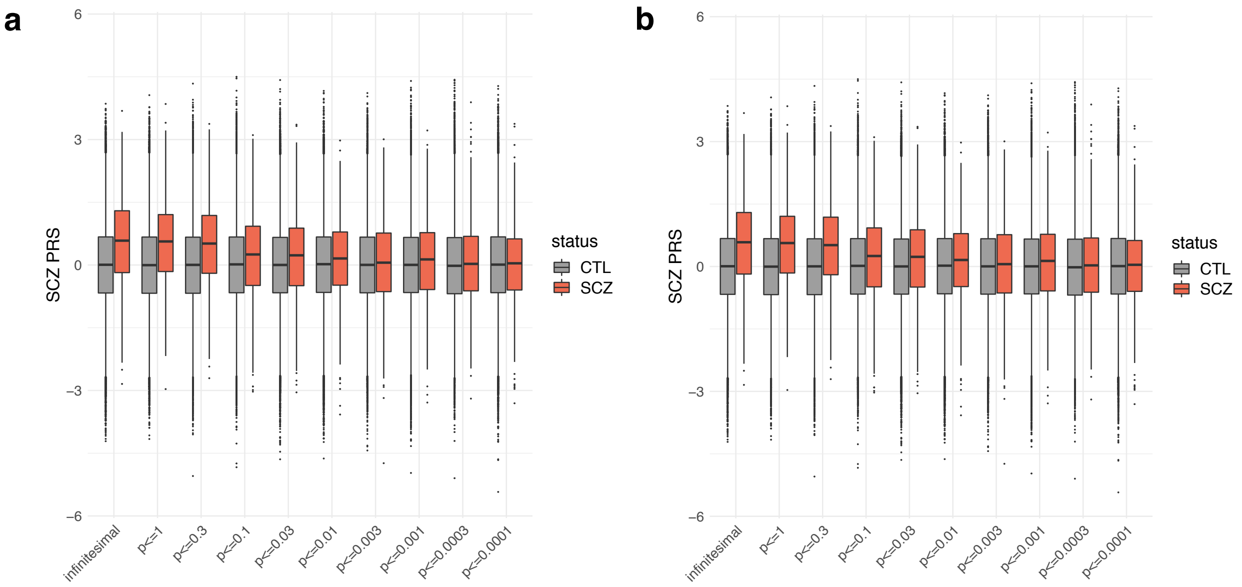

Supplementary Figure 4. Predictive ability of SCZ PRSs in EstBB. Boxplots of ten SCZ PRS values for SCZ cases and controls in EstBB testing set using either (a) wider diagnosis of schizophrenia spectrum disorder or (b) strictly schizophrenia cases.

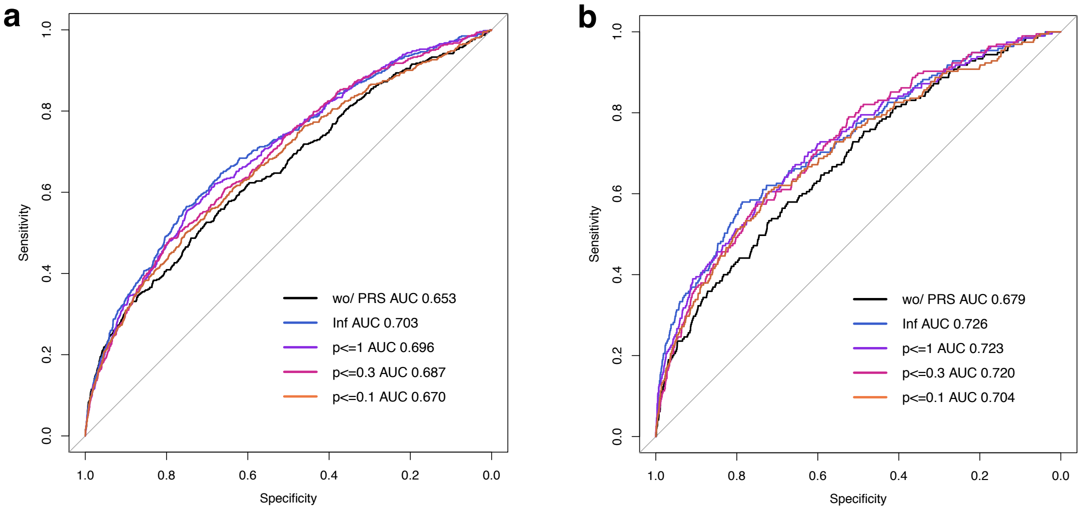

Supplementary Figure 5. Predictive ability of SCZ PRSs in EstBB. Discriminative capacity determined based on AUC in the EstBB validation set calculated for logistic regression models without and with SCZ PRS (four best performing SCZ PRSs are considered independently). Note that the x-axis is decreasing from 1 to 0 (exactly the same as if displaying “1 – specificity” increasing from 0 to 1). wo/ indicates a logistic regression model without SCZ PRS.

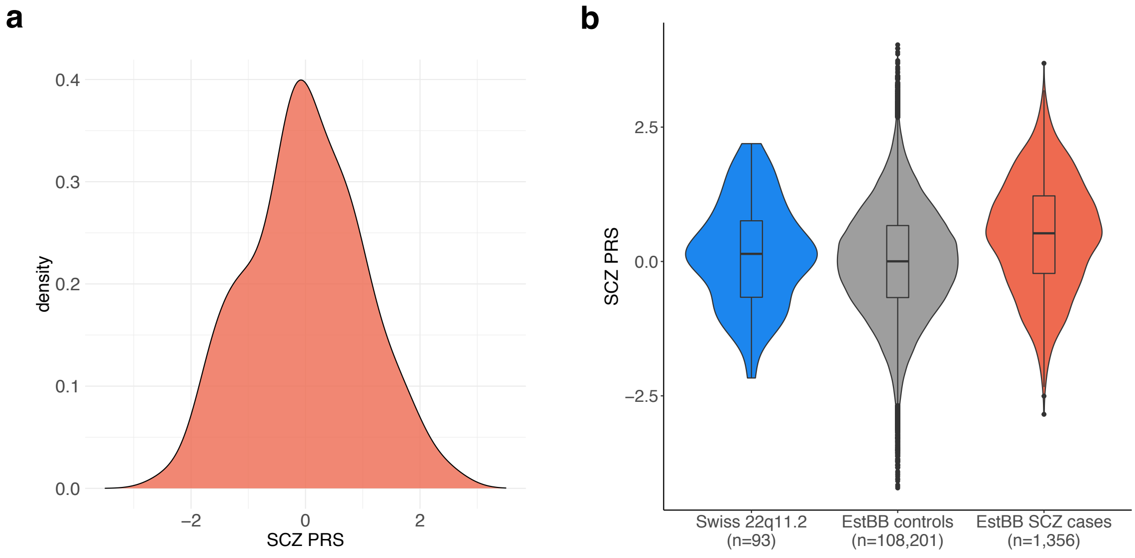

Supplementary Figure 6. Distribution of SCZ PRS values in the Swiss 22q11.2DS cohort and the Estonian Biobank (EstBB). (a) SCZ PRS for 22q11.2DS samples within the Swiss cohort is standardized such that it follows normal distribution with mean 0 and standard deviation 1. (b) Distribution of SCZ PRS values in the Swiss 22q11.2DS cohort in relation to control subjects and SCZ cases in EstBB.

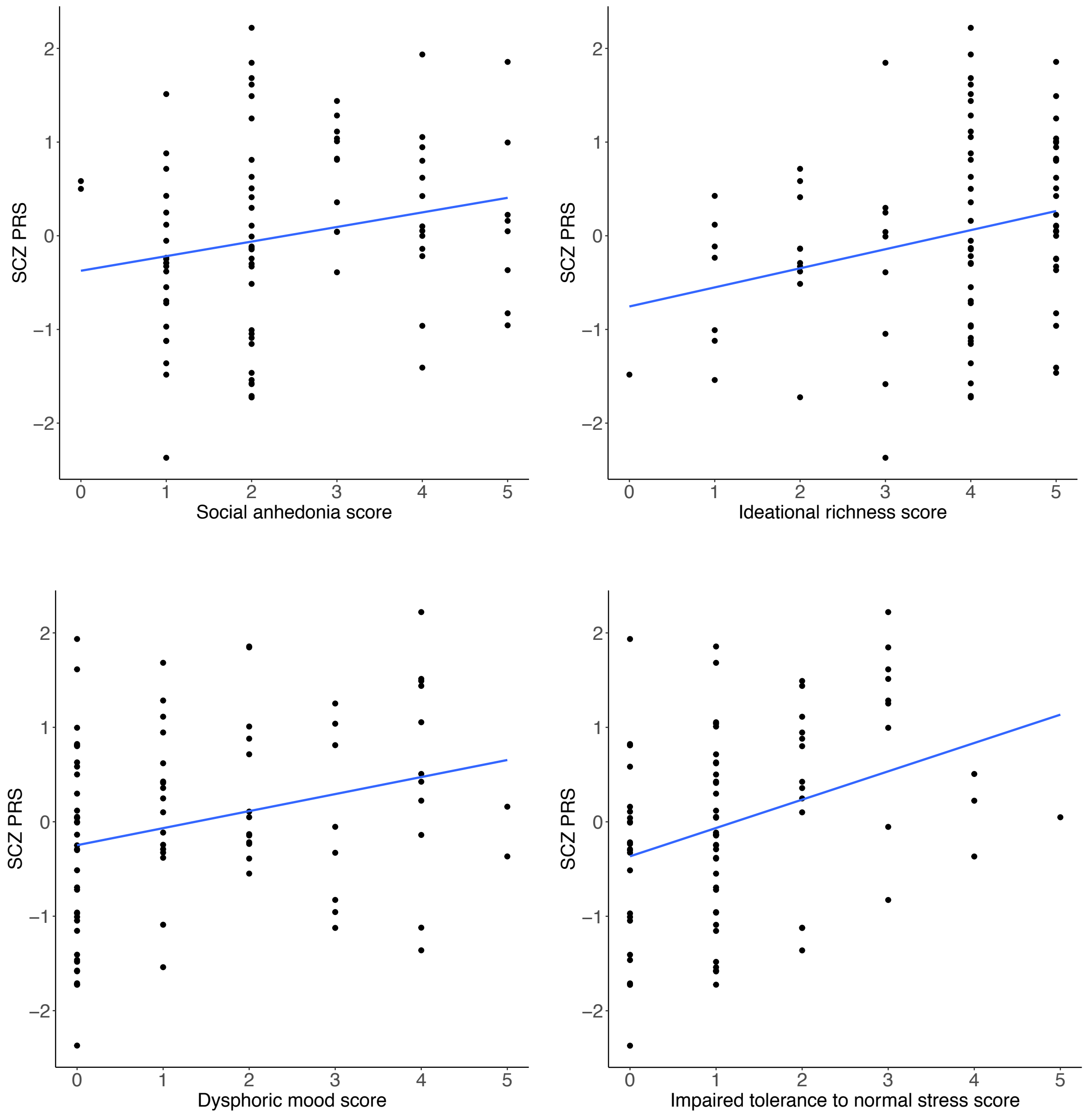

Supplementary Figure 7. Distribution of score values for SIPS variables determined as significant at FDR 10% in cross-sectional analysis as a function of standardized SCZ PRS.

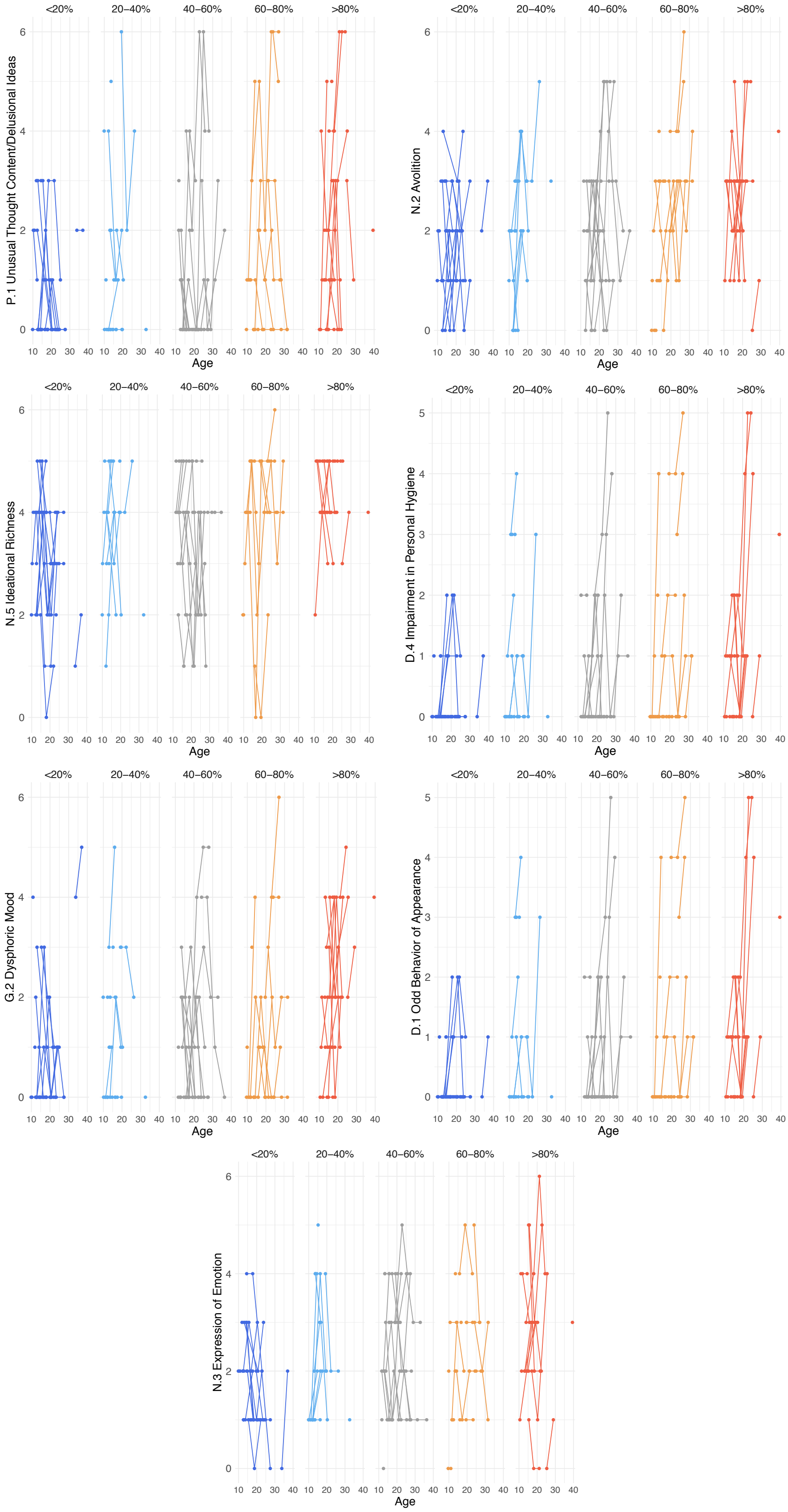

Supplementary Figure 8. Distributions of score values for SIPS variables over time and coloured by increasing SCZ PRS quintiles (dark blue, light blue, grey, orange, and red). Seven additional items that surpassed Bonferroni correction or ranked the highest based on bootstrapping in longitudinal analysis are outlined. Each dot represents a score determined at a given timepoint (visit) connected with a straight line for each 22q11.2DS patient.

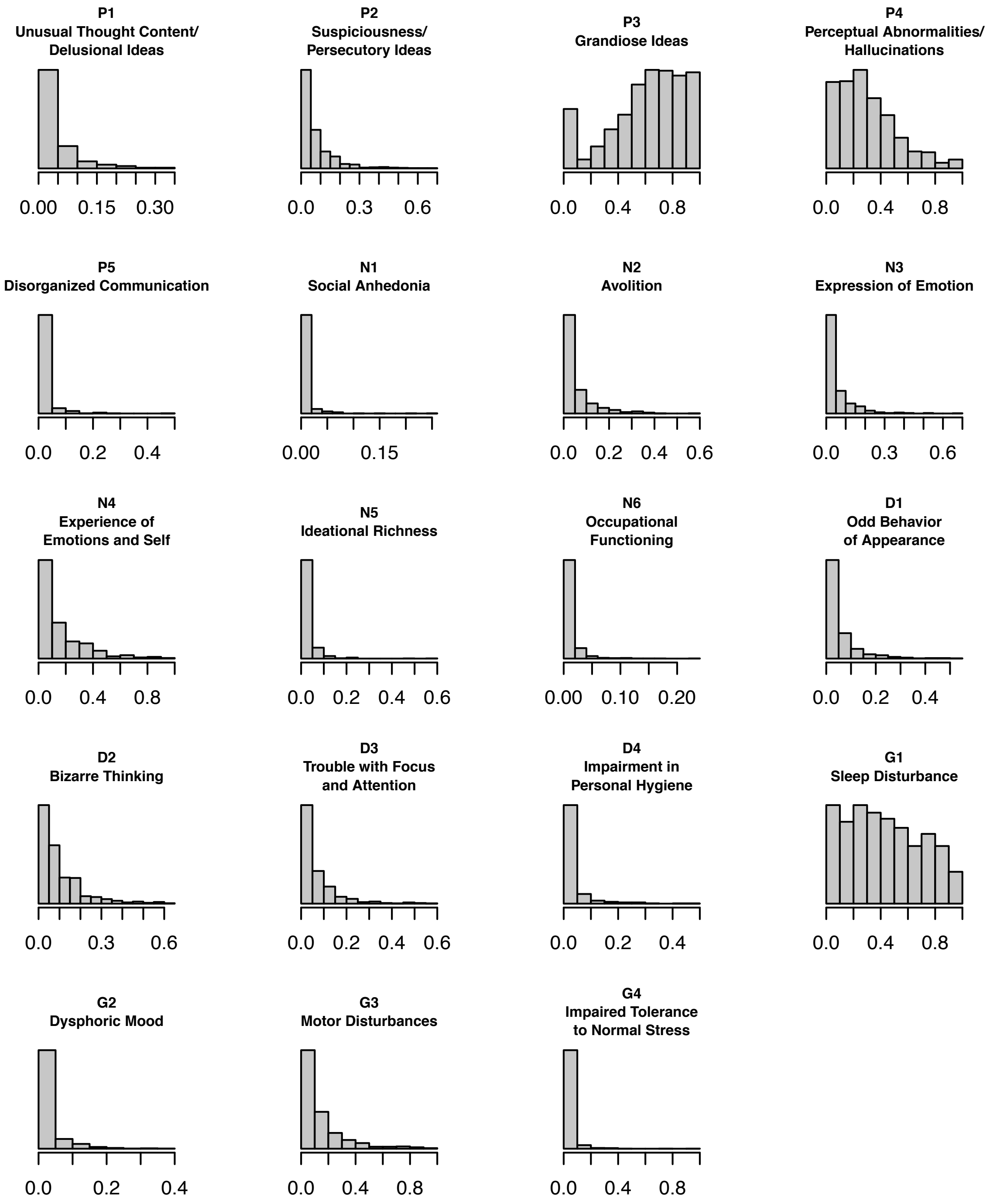

Supplementary Figure 9. Nominal p-values (x-axis) across bootstrapping runs for longitudinal association models with 19 SIPS variables.

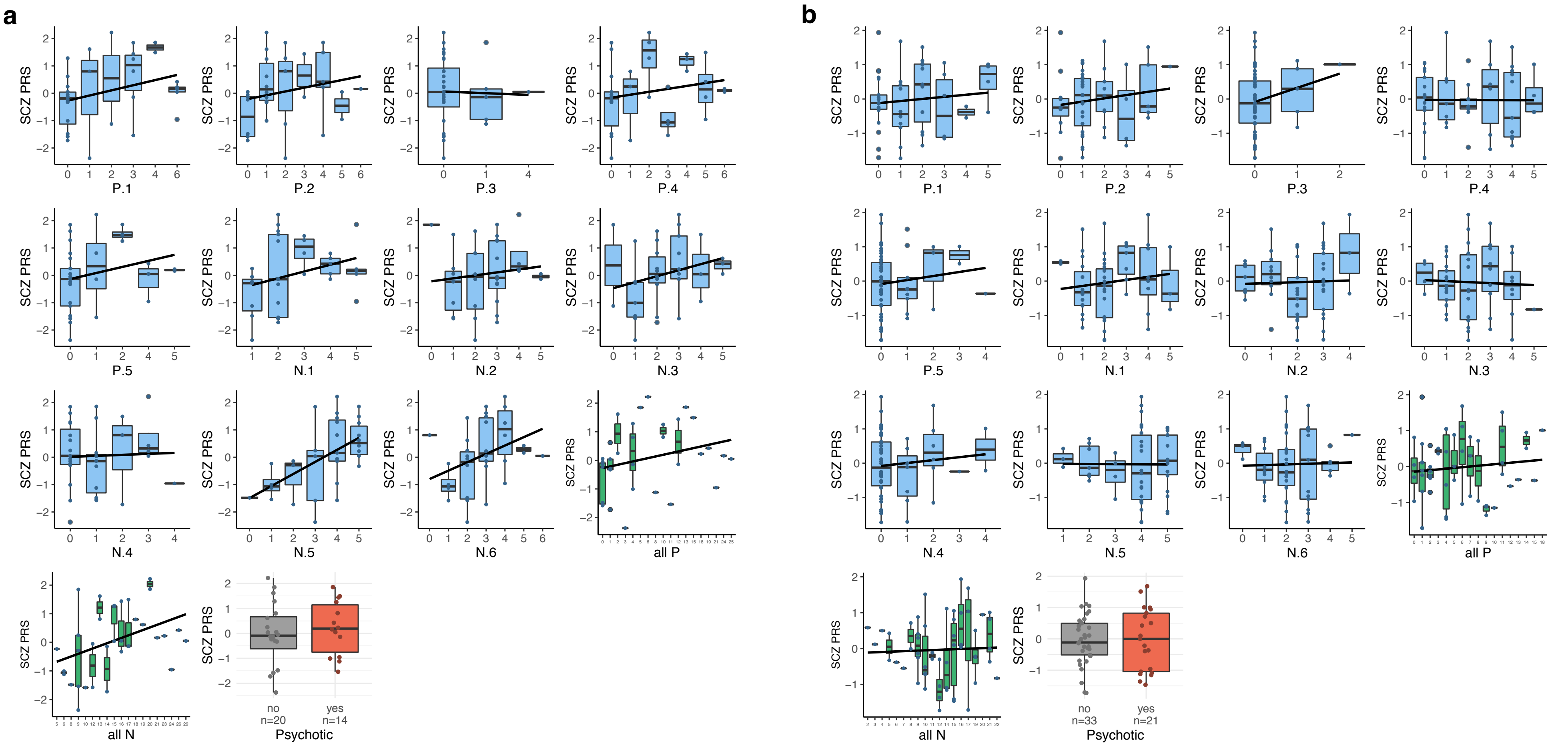

Supplementary Figure 10. Boxplots for score values for SIPS variables cross-sectionally. (a) displays score values captured before 18 years of age (n=34); (b) displays score values captured ≥18 years (n=54). Positive symptoms: P.1 – Unusual Thought Content/Delusional Ideas; P.2 – Suspiciousness/Persecutory Ideas; P.3 – Grandiose Ideas; P.4 – Perceptual Abnormalities/Hallucinations; P.5 – Disorganized Communication. Negative symptoms: N.1 – Social Anhedonia; N.2 – Avolition; N.3 – Expression of Emotion; N.4 – Experience of Emotions and Self; N.5 – Ideational Richness; N.6 – Occupational Functioning. all P – score values pooled across positive symptoms; all N – score values pooled across negative symptoms.

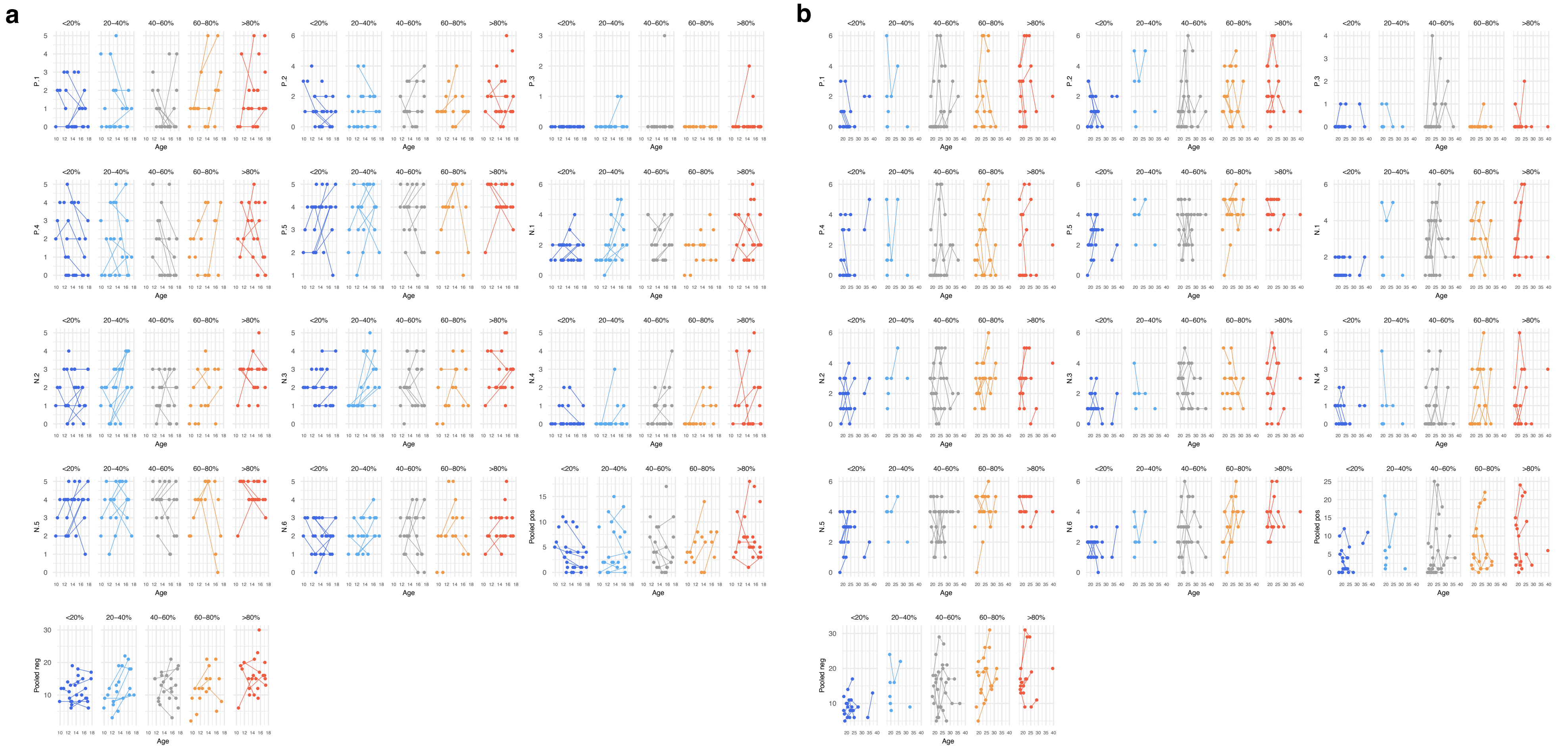

Supplementary Figure 11. Distributions of score values for SIPS variables over time and coloured by increasing SCZ PRS quintiles (dark blue, light blue, grey, orange, and red, respectively). (a) displays score values captured at timepoints before 18 years of age (n=76, 111 timepoints); (b) displays score values captured at timepoints $\geq$18 years (n=49, 102 timepoints). Each dot represents a score determined at a given timepoint (visit) connected with a straight line for each 22q11.2DS patient. Positive symptoms: P.1 – Unusual Thought Content/Delusional Ideas; P.2 – Suspiciousness/Persecutory Ideas; P.3 – Grandiose Ideas; P.4 – Perceptual Abnormalities/Hallucinations; P.5 – Disorganized Communication. Negative symptoms: N.1 – Social Anhedonia; N.2 – Avolition; N.3 – Expression of Emotion; N.4 – Experience of Emotions and Self; N.5 – Ideational Richness; N.6 – Occupational Functioning. Pooled pos – score values pooled across positive symptoms; Pooled neg – score values pooled across negative symptoms.

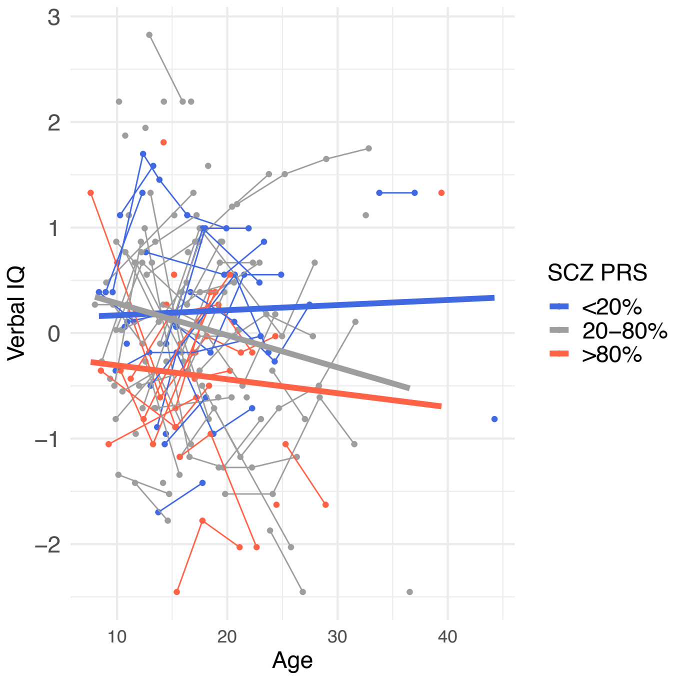

Supplementary Figure 12. Distribution of verbal IQ measurements across time for 22q11.2DS patients. Each dot denotes an IQ measurement determined at given timepoint (visit) connected by a straight line for each patient. The subjects are coloured based on their clustering on SCZ PRS distribution. The blue and red denote the lowest and the highest SCZ PRS quintile, respectively, with grey marking joint three middle quintiles.

Supplementary Table 1. Characteristics of the EstBB cohort used for testing and validating SCZ PRSs.

Cases with Schizophrenia Spectrum Disorder diagnosis (ICD-10 F2*)				Cases with strictly Schizophrenia diagnosis (ICD-10 F20.*)			
Sample	Feature	Test	Validation	Sample	Feature	Test	Validation
Control	n	71,412	36,789	Control	n	71,412	36,789
	Sex	M: 24,502; F: 46,910	M: 12,632; F: 24,157		Sex	M: 24,502; F: 46,910	M: 12,632; F: 24,157
	Age at last follow-up	47.74 (15.98)	47.62 (15.81)		Age at last follow-up	47.74 (15.98)	47.62 (15.81)
SCZ case	n	894	462	SCZ case	n	377	195
	Sex	M: 301; F: 593	M: 168; F: 294		Sex	M: 134; F: 243	M: 81; F: 114
	Age at diagnosis	39.00 (16.82)	39.19 (16.57)		Age at diagnosis	36.00 (13.32)	36.00 (14.35)

Two sub-group criteria were used for defining SCZ cases in EstBB: ICD-10 F2* for Schizophrenia Spectrum Disorder diagnosis group; and ICD-10 F20.* for Schizophrenia diagnosis group. Age is given in years with standard deviation in brackets. M denotes males and F females.

Supplementary Table 2. Association testing between SCZ PRS and SIPS variables cross-sectionally and longitudinally.

SIPS variable	Cross-sectional analysis				
**Positive symptoms**	**OR**	**95% CI**	**nominal p-value**	**FDR**	**Brant probability**
P.1 Unusual Thought Content/Delusional Ideas	1.37	0.94-2.01	0.11	0.261	0.76
P.2 Suspiciousness/Persecutory Ideas	1.39	0.94-2.08	0.11	0.261	0.96
P.3 Grandiose Ideas	1.11	0.56-2.21	0.76	0.813	0.96
P.4 Perceptual Abnormalities/Hallucinations	1.16	0.79-1.72	0.44	0.557	0.06
P.5 Disorganized Communication	1.60	1.00-2.64	0.06	0.222	0.98
**Negative symptoms**	**OR**	**95% CI**	**nominal p-value**	**FDR**	**Brant probability**
N.1 Social Anhedonia	1.61	1.08-2.43	0.02	0.095	0.75
N.2 Avolition	1.27	0.86-1.89	0.23	0.437	0.36
N.3 Expression of Emotion	1.21	0.83-1.78	0.31	0.491	0.32
N.4 Experience of Emotions and Self	1.04	0.68-1.59	0.86	0.86	0.91
N.5 Ideational Richness	1.69	1.14-2.54	0.01	0.063	0.98
N.6 Occupational Functioning	1.42	0.97-2.09	0.07	0.222	0.74
**Disorganization symptoms**	**OR**	**95% CI**	**nominal p-value**	**FDR**	**Brant probability**
D.1 Odd Behavior of Appearance	1.21	0.80-1.88	0.37	0.541	0.24
D.2 Bizarre Thinking	1.17	0.73-1.88	0.52	0.618	0.78
D.3 Trouble with Focus and Attention	1.32	0.89-1.98	0.17	0.359	0.14
D.4 Impairment in Personal Hygiene	1.25	0.83-1.88	0.28	0.484	0.54
**General symptoms**	**OR**	**95% CI**	**nominal p-value**	**FDR**	**Brant probability**
G.1 Sleep Disturbance	1.06	0.70-1.61	0.77	0.813	0.86
G.2 Dysphoric Mood	1.75	1.16-2.69	0.009	0.063	0.15
G.3 Motor Disturbances	1.20	0.76-1.92	0.43	0.557	0.98
G.4 Impaired Tolerance to Normal Stress	2.03	1.34-3.13	0.001	0.019	0.27

SIPS variable	Longitudinal analysis							
**Positive symptoms**	**OR**	**95% CI**	**nominal p-value**	**FDR**	**Bonferroni correction**	**mean p-value across 1000 runs**	**% of <0.05**	**model ranking**
P.1 Unusual Thought Content/Delusional Ideas	1.78	1.16-2.73	0.008	0.014	0.152	0.043	73.0	8
P.2 Suspiciousness/Persecutory Ideas	1.57	1.07-2.29	0.02	0.029	0.380	0.080	52.6	13
P.3 Grandiose Ideas	0.91	0.49-1.71	0.77	0.77	1.000	0.607	8.0	18
P.4 Perceptual Abnormalities/Hallucinations	1.34	0.86-2.09	0.20	0.22	1.000	0.308	8.1	17
P.5 Disorganized Communication	2.37	1.41-3.99	0.001	0.003	0.019	0.019	91.6	3
**Negative symptoms**	**OR**	**95% CI**	**nominal p-value**	**FDR**	**Bonferroni correction**	**mean p-value across 1000 runs**	**% of <0.05**	**model ranking**
N.1 Social Anhedonia	2.09	1.42-3.07	0.0002	0.002	0.004	0.006	98.1	1
N.2 Avolition	1.61	1.21-2.14	0.001	0.003	0.019	0.057	66.5	11
N.3 Expression of Emotion	1.81	1.17-2.81	0.008	0.014	0.152	0.054	67.5	10
N.4 Experience of Emotions and Self	1.47	1.01-2.14	0.04	0.049	0.798	0.153	32.8	16
N.5 Ideational Richness	1.78	1.22-2.60	0.003	0.007	0.057	0.025	86.2	5
N.6 Occupational Functioning	1.82	1.32-2.51	0.0003	0.002	0.006	0.010	96.6	2
**Disorganization symptoms**	**OR**	**95% CI**	**nominal p-value**	**FDR**	**Bonferroni correction**	**mean p-value across 1000 runs**	**% of <0.05**	**model ranking**
D.1 Odd Behavior of Appearance	2.00	1.20-3.33	0.008	0.014	0.152	0.051	68.4	9
D.2 Bizarre Thinking	1.93	1.08-3.42	0.025	0.033	0.475	0.093	43.4	14
D.3 Trouble with Focus and Attention	1.71	1.12-2.60	0.013	0.021	0.247	0.071	55.4	12
D.4 Impairment in Personal Hygiene	1.82	1.29-2.56	0.0007	0.003	0.013	0.027	85.7	7
**General symptoms**	**OR**	**95% CI**	**nominal p-value**	**FDR**	**Bonferroni correction**	**mean p-value across 1000 runs**	**% of <0.05**	**model ranking**
G.1 Sleep Disturbance	1.22	0.77-1.94	0.40	0.42	1.000	0.458	6.4	19
G.2 Dysphoric Mood	2.00	1.28-3.11	0.002	0.005	0.038	0.026	85.9	6
G.3 Motor Disturbances	1.64	1.05-2.55	0.028	0.035	0.532	0.131	37.6	15
G.4 Impaired Tolerance to Normal Stress	1.76	1.31-2.36	0.0002	0.002	0.004	0.024	89.1	4

In cross-sectional analysis, associations below FDR 10% were considered as significant (highlighted in grey). In longitudinal analysis, Bonferroni correction (accounting 19 tests) as well as validation using bootstrapping was applied. For bootstrapping analysis results, model ranking outlines significance rank across all models, which was retrieved by 1) calculating the proportion of associations p<0.05 across 1000 runs (% of <0.05) for each item and 2) ranking the proportions from highest to lowest across all models (model ranking). Significant associations that surpassed Bonferroni and that were supported by bootstrapping are highlighted in grey).

Supplementary Table 3. Association testing between SCZ PRS and SIPS items within positive and negative symptoms category in younger individuals vs older individuals cross-sectionally and longitudinally.

	Cross-sectional analysis								Longitudinal analysis									
SIPS variable	Below <18 (n=54)				Above >=18 (n=34)				Below <18 (n=76, 111 timepoints)					Above >=18 (n=49, 102 timepoints)				
**Positive symptoms**	**OR**	**95% CI**	**P**	**FDR**	**OR**	**95% CI**	**P**	**FDR**	**OR**	**95% CI**	**P**	**FDR**	**BF**	**OR**	**95% CI**	**P**	**FDR**	**BF**
P.1 Unusual Thought Content / Delusional Ideas	0.98	0.54-1.75	0.93	0.96	1.48	0.91-2.06	0.17	0.34	1.45	0.96-2.19	0.08	0.14	1.000	2.24	1.18-4.25	0.01	0.02	0.130
P.2 Suspiciousness / Persecutory Ideas	1.10	0.60-2.04	0.76	0.96	1.54	0.97-2.13	0.13	0.3	1.46	0.89-2.43	0.14	0.18	1.000	1.89	1.10-3.23	0.02	0.03	0.260
P.3 Grandiose Ideas	1.38	0.60-2.03	0.61	0.96	0.78	-0.14-0.93	0.61	0.71	NA*	NA*	NA*	NA*	NA*	0.86	0.46-1.59	0.63	0.63	1.000
P.4 Perceptual Abnormalities	0.92	0.51-1.66	0.78	0.96	1.19	0.63-1.74	0.53	0.66	1.56	0.98-2.49	0.06	0.12	0.780	1.30	0.642.65	0.47	0.51	1.000
P.5 Disorganized Communication	1.25	0.59-2.70	0.55	0.96	1.56	0.88-2.25	0.2	0.35	2.95	1.43-6.10	0.003	0.04	0.039	2.62	1.04-6.63	0.04	0.06	0.520
**Negative symptoms**	**OR**	**95% CI**	**P**	**FDR**	**OR**	**95% CI**	**P**	**FDR**	**OR**	**95% CI**	**P**	**FDR**	**BF**	**OR**	**95% CI**	**P**	**FDR**	**BF**
N.1 Social Anhedonia	1.48	0.83-2.66	0.18	0.96	2.16	0.74-1.86	0.02	0.09	1.59	0.99-2.55	0.06	0.12	0.780	3.42	1.72-6.80	0.0005	0.002	0.007
N.2 Avolition	1.12	0.60-2.09	0.72	0.96	1.30	0.74-1.86	0.36	0.5	1.55	1.05-2.29	0.03	0.09	0.390	1.72	1.09-2.70	0.02	0.03	0.260
N.3 Expression of Emotion	0.89	0.51-1.54	0.68	0.96	2.00	1.38-2.61	0.03	0.11	1.41	0.84-2.34	0.18	0.18	1.000	2.72	1.38-5.38	0.004	0.01	0.052
N.4 Experience of Emotions and Self	1.08	0.51-2.27	0.84	0.96	0.97	0.38-1.56	0.93	0.93	1.54	0.88-2.69	0.13	0.18	1.000	1.46	0.87-2.43	0.15	0.18	1.000
N.5 Ideational Richness	1.01	0.60-1.80	0.96	0.96	2.82	2.16-3.50	0.002	0.03	1.29	0.89-1.86	0.18	0.18	1.000	2.79	1.47-5.28	0.002	0.007	0.026
N.6 Occupational Functioning	0.98	0.55-1.76	0.96	0.96	1.96	1.39-2.53	0.02	0.09	1.33	0.89-2.00	0.17	0.18	1.000	2.24	1.46-3.42	0.0002	0.002	0.003
**Derived variables**	**β / OR**	**SE**	**P**	**FDR**	**β**	**SE**	**P**	**FDR**	**β**	**SE**	**P**	**FDR**	**BF**	**β / OR**	**SE**	**P**	**FDR**	**BF**
Pooled positive symptoms	0.20	0.72	0.76	0.96	1.03	1.21	0.23	0.34	0.25	0.10	0.018	0.09	0.234	0.21	0.11	0.06	0.08	0.780
Pooled negative symptoms	0.27	0.82	0.73	0.96	1.98	0.88	0.04	0.11	0.23	0.1	0.024	0.09	0.312	0.39	0.1	0.0005	0.002	0.007
Psychosis	0.88	0.42-1.81	0.74	0.96	1.03	0.51-2.09	0.92	0.93	NA*	NA*	NA*	NA*	NA*	NA*	NA*	NA*	NA*	NA*

Age distribution for the younger sample set (<18-year-old 22q11.2DS patients): mean 14.51, median 14.67, SD 2.16; and for the older sample set (≥18-year-old 22q11.2DS patients) mean 21.72, median 20.39, SD 4.81. Highlighted associations: in cross-sectional SIPS analysis, associations below FDR 10%; in longitudinal SIPS analysis, Bonferroni corrected (accounting for 13 tests) and associations below FDR 10%. P – nominal p-value; BF – Bonferroni correction. NA* - analysis could not be carried out due to low variability in response variable P3 and due to the categorization criteria of psychosis positive variable (criterion of lifetime presence of psychosis diagnosis, not separable for this analysis).

Supplementary Table 4. Association testing between SCZ PRS and IQ measurements cross-sectionally and longitudinally.

	Cross-sectional analysis			Longitudinal analysis			
**IQ variable**	β	**SE**	**nominal p-value**	β	**SE**	**nominal p-value**	**marginal R2**
Full scale IQ	-0.20	0.11	0.062	-0.25	0.11	0.020	0.13
verbal IQ	-0.23	0.11	0.038	-0.25	0.11	0.024	0.10
performance IQ	-0.14	0.11	0.183	-0.19	0.11	0.077	0.10

Marginal R2 denotes mean variance explained by fixed effects, i.e., expected variance explained for an independent dataset.

Supplementary Table 5. Association testing between SCZ PRS and brain imaging variables cross-sectionally and longitudinally.

	Cross-sectional analysis				Longitudinal analysis				
**Main MRI analysis**	β	**SE**	**nominal p-value**	**FDR**	β	**SE**	**nominal p-value**	**FDR**	**marginal R2**
Total cortical gray matter	-0.11	0.08	0.16	0.160	-0.12	0.08	0.112	0.112	0.47
Right hippocampus	-0.30	0.10	0.0038	0.010	-0.28	0.10	0.0047	0.014	0.15
Left hippocmapus	-0.28	0.10	0.010	0.014	-0.23	0.10	0.0169	0.025	0.17
**Hippocampus subfields**	β	**SE**	**nominal p-value**	**FDR**	β	**SE**	**nominal p-value**	**FDR**	**marginal R2**
Left tail	-0.23	0.11	0.028	0.187	-0.26	0.10	0.007	0.104	0.16
Left subiculum	-0.11	0.11	0.324	0.378	-0.12	0.10	0.216	0.274	0.1
Left CA1	-0.15	0.10	0.159	0.309	-0.14	0.10	0.132	0.231	0.13
Left molecular layer	-0.12	0.11	0.280	0.356	-0.12	0.10	0.235	0.274	0.11
Left GC-ML-DG	-0.08	0.10	0.424	0.424	-0.09	0.10	0.344	0.344	0.16
Left CA2/3	-0.17	0.10	0.100	0.280	-0.13	0.10	0.186	0.260	0.16
Left CA4	-0.10	0.11	0.352	0.379	-1.11	0.10	0.273	0.294	0.15
Right tail	-0.14	0.11	0.191	0.309	-0.18	0.10	0.088	0.176	0.16
Right subiculum	-0.14	0.11	0.200	0.309	-0.18	0.10	0.079	0.176	0.1
Right CA1	-0.22	0.10	0.038	0.187	-0.22	0.10	0.037	0.176	0.15
Right molecular layer	-0.16	0.11	0.133	0.309	-0.17	0.10	0.084	0.176	0.12
Right GC-ML-DG	-0.13	0.11	0.221	0.309	-0.14	0.10	0.164	0.255	0.15
Right CA2/3	-0.21	0.10	0.040	0.187	-0.19	0.10	0.055	0.176	0.2
Right CA4	-0.19	0.11	0.074	0.259	-0.2	0.10	0.053	0.176	0.14

In main MRI analysis, significant associations at FDR 5% are highlighted in grey. In hippocampal subfield analysis, associations at nominal p-value threshold ($p<0.05$) are highlighted in grey. Marginal R2 denotes mean variance explained by fixed effects, i.e., expected variance explained for an independent dataset.

Supplementary Table 6. Association testing between SCZ PRS and all considered neuropsychiatric phenotypes with a sample set without 5 individuals carrying 1.5Mb deletion.

SIPS variable	Cross-sectional analysis				Longitudinal analysis					
**Positive symptoms**	**OR**	**95% CI**	**nominal p-value**	**FDR**	**OR**	**95% CI**	**nominal p-value**	**FDR**	**Bonferroni**	**mean p-value across 1000 runs**
P.1 Unusual Thought Content/Delusional Ideas	1.34	0.91-1.02	0.15	0.36	1.76	1.15-2.70	0.009	0.019	0.171	0.044
P.2 Suspiciousness/Persecutory Ideas	1.34	0.89-2.05	0.17	0.36	1.57	1.06-2.31	0.020	0.029	0.380	0.088
P.3 Grandiose Ideas	1.30	0.62-2.81	0.49	0.68	0.91	0.46-1.77	0.780	0.780	1.000	0.625
P.4 Perceptual Abnormalities/Hallucinations	1.15	0.77-1.72	0.50	0.68	1.33	0.85-2.08	0.220	0.246	1.000	0.340
P.5 Disorganized Communication	1.48	0.92-2.47	0.11	0.36	2.38	1.40-4.06	0.001	0.003	0.019	0.018
**Negative symptoms**	**OR**	**95% CI**	**nominal p-value**	**FDR**	**OR**	**95% CI**	**nominal p-value**	**FDR**	**Bonferroni**	**mean p-value across 1000 runs**
N.1 Social Anhedonia	1.48	0.98-2.24	0.06	0.29	1.97	1.34-2.88	0.0005	0.003	0.001	0.013
N.2 Avolition	1.24	0.83-1.86	0.30	0.57	1.64	1.23-2.17	0.0006	0.003	0.011	0.052
N.3 Expression of Emotion	1.09	0.74-1.61	0.64	0.76	1.74	1.10-2.74	0.018	0.029	0.342	0.080
N.4 Experience of Emotions and Self	1.00	0.64-1.57	0.98	0.98	1.49	1.02-2.19	0.040	0.046	0.760	0.145
N.5 Ideational Richness	1.54	1.03-2.32	0.04	0.25	1.59	1.10-2.30	0.014	0.027	0.266	0.065
N.6 Occupational Functioning	1.31	0.89-1.93	0.17	0.36	1.73	1.26-2.39	0.0008	0.003	0.015	0.018
**Disorganization symptoms**	**OR**	**95% CI**	**nominal p-value**	**FDR**	**OR**	**95% CI**	**nominal p-value**	**FDR**	**Bonferroni**	**mean p-value across 1000 runs**
D.1 Odd Behavior of Appearance	1.11	0.72-1.73	0.63	0.76	1.85	1.11-3.08	0.018	0.029	0.342	0.082
D.2 Bizarre Thinking	1.05	0.65-1.70	0.84	0.89	1.87	1.04-3.38	0.038	0.048	0.722	0.115
D.3 Trouble with Focus and Attention	1.39	0.92-2.12	0.12	0.36	1.77	1.17-2.69	0.007	0.017	0.133	0.058
D.4 Impairment in Personal Hygiene	1.20	0.79-1.83	0.39	0.67	1.78	1.25-2.52	0.001	0.003	0.019	0.034
**General symptoms**	**OR**	**95% CI**	**nominal p-value**	**FDR**	**OR**	**95% CI**	**nominal p-value**	**FDR**	**Bonferroni**	**mean p-value across 1000 runs**
G.1 Sleep Disturbance	1.05	0.69-1.60	0.81	0.89	1.17	0.74-1.85	0.500	0.530	1.000	0.511
G.2 Dysphoric Mood	1.77	1.15-2.76	0.01	0.10	2.07	1.33-3.21	0.001	0.003	0.019	0.020
G.3 Motor Disturbances	1.18	0.73-1.93	0.50	0.67	1.62	1.04-2.51	0.031	0.042	0.589	0.117
G.4 Impaired Tolerance to Normal Stress	2.09	1.35-3.31	0.001	0.02	1.78	1.33-2.38	0.0001	0.002	0.002	0.018

IQ variables	β	SE	nominal p-value
Full scale IQ	-0.26	0.11	0.019
verbal IQ	-0.24	0.11	0.029
performance IQ	-0.21	0.11	0.055

Main MRI analysis	β	SE	nominal p-value	FDR
Total cortical gray matter	-0.12	0.08	0.134	0.134
Right hippocampus	-0.26	0.1	0.007	0.021
Left hippocmapus	-0.21	0.1	0.025	0.038
**Hippocampus subfields**	β	**SE**	**nominal p-value**	**FDR**
Left tail	-0.25	0.1	0.012	0.168
Left subiculum	-0.11	0.1	0.225	0.293
Left CA1	-0.14	0.1	0.145	0.254
Left molecular layer	-0.10	0.1	0.290	0.338
Left GC-ML-DG	-0.08	0.1	0.392	0.392
Left CA2/3	-0.12	0.1	0.230	0.293
Left CA4	-0.10	0.1	0.325	0.350
Right tail	-0.17	0.1	0.106	0.232
Right subiculum	-0.16	0.1	0.108	0.232
Right CA1	-0.21	0.1	0.055	0.232
Right molecular layer	-0.16	0.1	0.116	0.232
Right GC-ML-DG	-0.13	0.1	0.198	0.293
Right CA2/3	-0.18	0.1	0.072	0.232
Right CA4	-0.20	0.1	0.067	0.232

Highlighted associations: in cross-sectional SIPS analysis, associations below FDR 10%; in longitudinal SIPS analysis, Bonferroni corrected (accounting for 19 tests); in MRI main analysis, associations below FDR 5%; in hippocampal subfield analysis, associations at nominal significance.

Supplementary Table 7. Association testing between SCZ PRS and all brain imaging variables according to the Desikan Killiany atlas longitudinally.

Volume	β	SE	p-value
Left_Lateral_Ventricle	-0.113	0.10	0.264
Left_Inf_Lat_Vent	-0.076	0.10	0.467
Left_Cerebellum_Cortex	-0.080	0.09	0.351
Left_Thalamus_Proper	-0.073	0.09	0.412
Left_Caudate	-0.062	0.09	0.501
Left_Putamen	0.004	0.09	0.966
Left_Pallidum	-0.049	0.09	0.568
Left_Hippocmapus	-0.225	0.10	0.0169
Third_Ventricle	-0.155	0.09	0.092
Fourth_Ventricle	-0.185	0.10	0.066
Brain_Stem	-0.017	0.09	0.846
Left_Amygdala	-0.021	0.09	0.804
CSF	0.034	0.10	0.734
Left_Accumbens_Area	0.002	0.09	0.984
Left_Ventral_Diancephalon	-0.053	0.09	0.544
Right_Lateral_Ventricle	-0.088	0.10	0.386
Right_Inf_Lat_Vent	-0.193	0.10	0.064
Right_Cerebellum_Cortex	-0.098	0.08	0.233
Right_Thalamus_Proper	-0.081	0.09	0.377
Right_Caudate	0.007	0.09	0.940
Right_Putamen	0.000	0.09	0.995
Right_Pallidum	-0.050	0.09	0.557
Right_Hippocampus	-0.277	0.10	0.005
Right_Amygdala	-0.110	0.09	0.221
Right_Accumbens_Area	0.002	0.09	0.978
Right_Ventral_Diancephalon	-0.046	0.09	0.609
Fifth_Ventricle	-0.005	0.07	0.949
lhCortexVol	-0.130	0.08	0.100
rhCortexVol	-0.121	0.08	0.122
TotalCorticalVol	-0.123	0.08	0.112
SubCortGrayVol	0.033	0.08	0.693
TotalGrayVol	-0.121	0.08	0.125
SupraTentorialVol	-0.097	0.09	0.263

Surface area	β	SE	p-value
lh_bankssts_area	-0.183	0.100	0.073
lh_caudalanteriorcingulate_area	0.056	0.099	0.569
lh_caudalmiddlefrontal_area	-0.153	0.102	0.135
lh_cuneus_area	-0.182	0.096	0.074
lh_entorhinal_area	-0.079	0.096	0.421
lh_fusiform_area	0.029	0.094	0.742
lh_inferiorparietal_area	-0.002	0.092	0.998
lh_inferiortemporal_area	0.009	0.092	0.906

lh_isthmuscingulate_area	-0.050	0.101	0.624
lh_lateraloccipital_area	-0.148	0.089	0.104
lh_lateralorbitofrontal_area	-0.089	0.096	0.354
lh_lingual_area	-0.023	0.097	0.855
lh_medialorbitofrontal_area	-0.066	0.087	0.474
lh_middletemporal_area	-0.153	0.089	0.098
lh_parahippocampal_area	0.011	0.100	0.909
lh_paracentral_area	-0.202	0.094	0.035
lh_parsopercularis_area	0.072	0.104	0.479
lh_parsorbitalis_area	-0.117	0.091	0.203
lh_parstriangularis_area	0.029	0.099	0.769
lh_pericalcarine_area	-0.046	0.102	0.682
lh_postcentral_area	-0.120	0.089	0.179
lh_posteriorcingulate_area	0.006	0.103	0.953
lh_precentral_area	-0.209	0.083	0.013
lh_precuneus_area	-0.037	0.100	0.714
lh_rostralanteriorcingulate_area	-0.025	0.091	0.805
lh_rostralmiddlefrontal_area	-0.169	0.090	0.062
lh_superiorfrontal_area	-0.175	0.092	0.062
lh_superiorparietal_area	-0.022	0.095	0.797
lh_superiortemporal_area	-0.178	0.099	0.075
lh_supramarginal_area	-0.056	0.096	0.553
lh_frontalpole_area	-0.126	0.090	0.165
lh_temporalpole_area	0.014	0.084	0.818
lh_transversetemporal_area	0.123	0.105	0.247
lh_insula_area	-0.065	0.095	0.507
rh_bankssts_area	-0.133	0.094	0.169
rh_caudalanteriorcingulate_area	-0.121	0.100	0.232
rh_caudalmiddlefrontal_area	-0.143	0.103	0.169
rh_cuneus_area	-0.096	0.095	0.326
rh_entorhinal_area	-0.041	0.094	0.656
rh_fusiform_area	-0.029	0.089	0.766
rh_inferiorparietal_area	-0.026	0.095	0.798
rh_inferiortemporal_area	-0.013	0.096	0.915
rh_isthmuscingulate_area	-0.067	0.098	0.493
rh_lateraloccipital_area	-0.064	0.088	0.489
rh_lateralorbitofrontal_area	0.015	0.095	0.878
rh_lingual_area	-0.042	0.103	0.701
rh_medialorbitofrontal_area	-0.008	0.090	0.951
rh_middletemporal_area	-0.109	0.091	0.238
rh_parahippocampal_area	-0.001	0.101	0.996
rh_paracentral_area	-0.157	0.091	0.088
rh_parsopercularis_area	-0.115	0.097	0.261
rh_parsorbitalis_area	-0.154	0.090	0.096
rh_parstriangularis_area	-0.175	0.097	0.083
rh_pericalcarine_area	-0.068	0.102	0.524

rh_postcentral_area	-0.116	0.088	0.192
rh_posteriorcingulate_area	-0.055	0.102	0.591
rh_precentral_area	-0.183	0.087	0.038
rh_precuneus_area	-0.044	0.094	0.642
rh_rostralanteriorcingulate_area	-0.114	0.087	0.199
rh_rostralmiddlefrontal_area	-0.112	0.087	0.202
rh_superiorfrontal_area	-0.046	0.095	0.636
rh_superiorparietal_area	-0.080	0.100	0.429
rh_superiortemporal_area	-0.195	0.090	0.031
rh_supramarginal_area	-0.009	0.094	0.919
rh_frontalpole_area	-0.086	0.090	0.345
rh_temporalpole_area	-0.003	0.094	0.985
rh_transversetemporal_area	-0.035	0.097	0.716
rh_insula_area	-0.110	0.092	0.231

**Thickness**	β	**SE**	**p-value**
lh_bankssts_thickness	0.016	0.094	0.868
lh_caudalanteriorcingulate_thickness	-0.055	0.092	0.550
lh_caudalmiddlefrontal_thickness	0.002	0.092	0.980
lh_cuneus_thickness	-0.048	0.088	0.591
lh_entorhinal_thickness	0.026	0.096	0.784
lh_fusiform_thickness	-0.094	0.082	0.260
lh_inferiorparietal_thickness	-0.007	0.080	0.933
lh_inferiortemporal_thickness	-0.086	0.080	0.285
lh_isthmuscingulate_thickness	-0.007	0.079	0.926
lh_lateraloccipital_thickness	-0.104	0.092	0.260
lh_lateralorbitofrontal_thickness	-0.065	0.081	0.425
lh_lingual_thickness	0.007	0.090	0.935
lh_medialorbitofrontal_thickness	-0.006	0.079	0.945
lh_middletemporal_thickness	-0.036	0.090	0.693
lh_parahippocampal_thickness	-0.030	0.101	0.769
lh_paracentral_thickness	0.028	0.095	0.771
lh_parsopercularis_thickness	-0.029	0.090	0.746
lh_parsorbitalis_thickness	-0.013	0.095	0.891
lh_parstriangularis_thickness	0.002	0.079	0.978
lh_pericalcarine_thickness	-0.110	0.088	0.217
lh_postcentral_thickness	0.023	0.092	0.806
lh_posteriorcingulate_thickness	0.012	0.085	0.890
lh_precentral_thickness	0.001	0.099	0.995
lh_precuneus_thickness	-0.001	0.082	0.988
lh_rostralanteriorcingulate_thickness	0.023	0.087	0.788
lh_rostralmiddlefrontal_thickness	-0.011	0.079	0.891
lh_superiorfrontal_thickness	-0.017	0.082	0.839
lh_superiorparietal_thickness	-0.024	0.084	0.774
lh_superiortemporal_thickness	0.007	0.098	0.945
lh_supramarginal_thickness	0.014	0.087	0.875
lh_frontalpole_thickness	0.017	0.089	0.849

lh_temporalpole_thickness	-0.026	0.089	0.769
lh_transversetemporal_thickness	-0.045	0.107	0.676
lh_insula_thickness	-0.082	0.085	0.336
rh_bankssts_thickness	0.031	0.105	0.771
rh_caudalanteriorcingulate_thickness	-0.043	0.095	0.654
rh_caudalmiddlefrontal_thickness	-0.062	0.091	0.500
rh_cuneus_thickness	0.002	0.087	0.982
rh_entorhinal_thickness	-0.039	0.089	0.663
rh_fusiform_thickness	-0.091	0.085	0.285
rh_inferiorparietal_thickness	-0.035	0.083	0.677
rh_inferiortemporal_thickness	-0.082	0.080	0.310
rh_isthmuscingulate_thickness	-0.034	0.087	0.698
rh_lateraloccipital_thickness	-0.088	0.088	0.322
rh_lateralorbitofrontal_thickness	-0.058	0.083	0.489
rh_lingual_thickness	-0.054	0.088	0.539
rh_medialorbitofrontal_thickness	-0.076	0.077	0.331
rh_middletemporal_thickness	0.038	0.089	0.675
rh_parahippocampal_thickness	-0.202	0.104	0.057
rh_paracentral_thickness	-0.025	0.096	0.798
rh_parsopercularis_thickness	-0.001	0.087	0.987
rh_parsorbitalis_thickness	-0.044	0.092	0.636
rh_parstriangularis_thickness	-0.006	0.082	0.941
rh_pericalcarine_thickness	-0.046	0.091	0.614
rh_postcentral_thickness	0.064	0.094	0.500
rh_posteriorcingulate_thickness	-0.029	0.079	0.719
rh_precentral_thickness	0.006	0.098	0.951
rh_precuneus_thickness	-0.005	0.083	0.952
rh_rostralanteriorcingulate_thickness	-0.150	0.083	0.076
rh_rostralmiddlefrontal_thickness	-0.001	0.071	0.993
rh_superiorfrontal_thickness	-0.057	0.082	0.489
rh_superiorparietal_thickness	-0.024	0.084	0.774
rh_superiortemporal_thickness	0.021	0.101	0.838
rh_supramarginal_thickness	0.003	0.091	0.975
rh_frontalpole_thickness	0.091	0.093	0.331
rh_temporalpole_thickness	0.072	0.088	0.418
rh_transversetemporal_thickness	-0.007	0.094	0.938
rh_insula_thickness	-0.119	0.086	0.168

Association testing results using linear mixed modelling is given for volume, thickness, and surface area measurements according to the Desikan Killiany atlas. All considered MRI measurements were regressed on SCZ PRS, age, age^2, MRI scanner and first three genotype PCs. Associations at nominal significance ($p<0.05$) are highlighted in grey.
